# Supplementary material for: The non-opponent nature of colour afterimages
Source: Commun Psychol. 2025 Nov 1;3:154. doi: 10.1038/s44271-025-00331-5 (PMC12579601; doi:10.1038/s44271-025-00331-5)
Supplement: Supplementary file 2 — Supplementary Material [file 44271_2025_331_MOESM2_ESM.pdf]

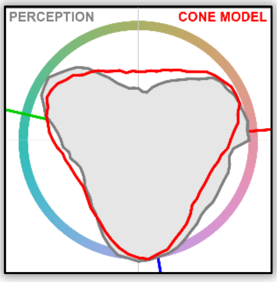

Supplementary Material

The Non-Opponent Nature of Colour Afterimages

Communications Psychology | 2025 | https://doi.org/10.1038/s44271-025-00331-5
Christoph Witzel | School of Psychology | University of Southampton | Southampton, UK
c.witzel@soton.ac.uk

Additional data, tables and code from this supplementary material are also available on Zenodo + Github

Table of Content

A. Animated Visualisations (Figure S1, Table S1) 2
1. Illustration of Afterimages 2
2. Illustration of the Method in Experiments 2-3 3
3. Illustration of Main Results 3
B. Experiment 1: Fixed-Location Task (Figures S2-S3, Table S2-S3) 4
1. Colour Specifications and Results per Inducer (Exp 1) 4
2. Details on Afterimage Measurements (Exp 1) 4
3. Control Task (Exp 1) 5
4. Colour Naming (Exp 1) 5
5. Additional Results from Experiment 1.a 7
C. Experiment 2: Chaser-Like Task (Figures S4-S12, Table S4) 7
1. Details on Participants and Apparatus (Exp 2) 7
2. Data Distributions (Exp 2) 8
3. Experiment 2.a: Results on Individual Observers 10
4. Experiment 2.b: Sampling in DKL Space 11
D. Experiment 3: Effects of Inducer Chroma on Afterimage Hue (Figure S13, Table S5) 12
1. Details on Participants and Apparatus (Exp 3) 12
2. Additional Results on the Effect of Inducer Chroma on Afterimage Hue (Exp 3) 13
E. Models (Figure S14) 14
1. Cone-Contrast Model 14
2. Cone-Opponent Model (DKL) 16
3. CIELUV 19
4. CIELAB 19
5. CIECAM02 19
6. Munsell-Opponent Model 20
7. Hering-Opponent Model 21

A. Animated Visualisations (Figure S1, Table S1)

Animated visualisations of colour afterimages are attached separately as *Supplementary Movies* in gif format. The colours have been rendered assuming a standard RGB (*sRGB*) display. Gif-format has been chosen to avoid the changes of RGB values as in other video formats. The animations illustrate in first-person experience (1) the phenomenon of complementary colour afterimages, (2) the task in *Experiments 2-3*, and (3) the key finding of this study. The static images in *Figure S1* give an overview of the four *Supplementary Movies*; *Table S1* provides corresponding colour specifications.

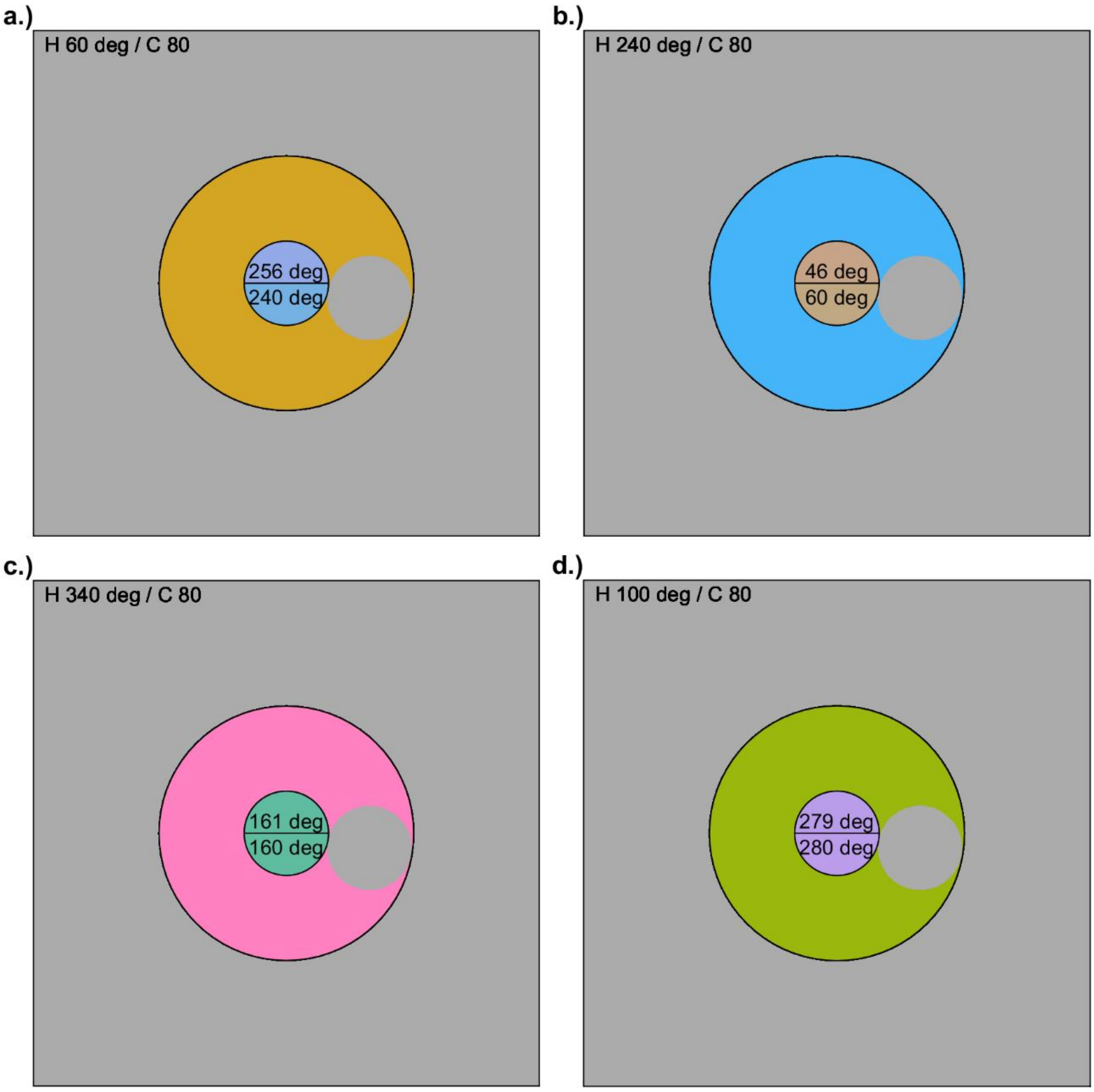

Figure S1 | Overview of Animated Visualisations (Supplementary Movies). All Animations are attached to the article as *Supplementary Movies 1-4* in gif-format. The figure shows static screenshots of those animations to provide an overview of the attachments. It needs at least 3 cycles for the afterimage to fully build up and produce the cone-adapted hue in the upper half. When the afterimage is weak, its hue is similar to the opponent hue, see *Experiment 3*. *Supplementary Movies 1-2* in panels a and b show examples, in which the prediction by the cone-adaptation model (lower half of centre disk) differs from the cone-opponent colour (upper half). The afterimage is expected to look like the upper rather than the lower half of the centre disk. *Supplementary Movies 3-4* in panels c and d provide contrary examples, in which cone-adaptation and cone-opponency predict the same afterimage hue, considering that a numerical difference of ~1 deg is too small to be visible. The afterimages in *Supplementary Movies 3-4* are thus expected to look like both halves of a unified centre circle. Colour specifications for reproduction are provided in *Table S1*.

1. Illustration of Afterimages

While traditionally afterimages are shown through the method explained in the *Introduction* of the main article, these animations provide an alternative, more stable way to visualise afterimages. In each animation, a grey disk is rotating around a coloured ring (cf. *Figure 2.e*). If you pursue the moving disk, you might be able to see it as grey. To see the afterimages, you should not pursue the disk. Instead, you need to fixate the cross at the centre for several seconds. As a rule of thumb, fixate for at least 3 cycles of the moving disk with firm centre fixation. When you keep fixating, the moving disk appears in an illusory colour; that colour is the complementary afterimage. The animation in *Supplementary Movie 1* provides the example with the yellowish inducer that has been elaborated in the main article. After fixating for several seconds, the moving disk will appear in a blue-purple colour, which is complementary to the yellowish inducer.

2. Illustration of the Method in Experiments 2-3

When the moving disk passes through the bottom-right quarter of the inducing ring, two comparison colours are shown in the upper and lower half of the centre disk. If your monitor is approximately standard (*sRGB*), you should be able to see differences between the half circles in *Supplementary Movies 1-2*. You are supposed to compare those to the afterimages that appear on the moving circle when fixating the centre fixation cross for a sustained time. However, note that, besides monitor calibration, there may also be differences across observers that modulate the strength of afterimages perceived on the moving circle (cf. individual results in *Figures S8-S10*). In this simplified visualisation, you can try to judge which of the two half-discs match more closely the illusory colour of the moving disk. This kind of perceptual comparison is the task in *Experiments 2 and 3 (cf. Figure 2.a)*. However, there weren't two half-disks, but only a single disk at the centre in the task of *Experiments 2 and 3*. Participants could adjust the colour of the centre disk to match the illusory colour of the moving disk.

3. Illustration of Main Results

The deviation of afterimage hue from cone opponency is so strong that it can be seen in *Supplementary Movies 1-2* on a standard monitor. In *Supplementary Movie 1*, the colour of the ring corresponds to the yellowish example inducer from *Figure 1* and *Figure 2.e*. It has a hue of 60 degrees and produces a particularly strong deviation from cone-opponency *(cf. Figure 2.g)*. The lower half presents the cone-opponent blue colour with a hue of 240 deg; the upper half displays the hue predicted by cone-adaptation. The perceived afterimage is visibly more purple than the cone-opponent colour. It should appear more similar to the upper than the lower half, or even the same colour as the upper half when fully adapted. Another example for the discrepancy between afterimage and cone-opponency is shown in *Supplementary Movie 2* with the inducer at 240 deg, thus cone-opponent to the first one (*Figure S1.b*).

For comparison, the inducer at 340 deg in *Supplementary Movie 3* and *Figure S1.c* provide an example where cone-adaptation predicts the same hue as cone-opponency *(cf. Figure 2.g)*. Thus, the moving afterimage in *Supplementary Movie 3* has the same hue as both halves appearing in the centre. *Supplementary Movie 4* with the inducer at 100 degree provides another example of this kind where cone-opponency and cone-adaptation predict the same hue (*Figure S1.d*).

Table S1 | Colour specifications of Animated Visualisations. Rows correspond to the different colour elements in each animation, where cone adaptation refers to the upper half-disks, and cone-opponency to the lower half-disks in the animations. Columns report polar coordinates in CIELUV, chromaticity coordinates and luminance in cd/m² (*xyY*), and standard RGB (*sRGB*). Inducer hues are emphasised by bold font. The last column visualises the sRGB values; these are the same as the colours in *Figure S1*.

	CIELUV			*CIE1931*			*sRGB*			
	*L**	*H [deg]*	*C*	*x*	*y*	*Y*	*R*	*G*	*B*	
Background	70	-	0	0.31238	.32852	32.6	171	171	171	
Animation 1 (*Supplementary Movie 1, Figure S1.a*)										
Inducer Ring	70	**60**	80	.45862	.45881	32.6	212	164	35	
Cone Adaptation	69.79	256.26	55.737	.25111	.24877	32.361	148	169	231	
Cone Opponency	70	240	55.737	.23643	.26085	32.6	117	177	226	
Animation 2 (*Supplementary Movie 2, Figure S1.b*)										
Inducer Ring	70	**240**	80	.2081	.23562	32.6	69	180	249	
Cone Adaptation	69.944	45.915	34.484	.37218	.36544	32.536	199	164	136	
Cone Opponency	70	60	34.484	.36894	.37891	32.6	192	168	130	
Animation 3 (*Supplementary Movie 3, Figure S1.c*)										
Inducer Ring	70	**340**	80	.37805	.26243	32.6	255	129	194	
Cone Adaptation	70.13	161.33	43.45	.26502	.37294	32.748	95	188	160	
Cone Opponency	70	160	43.45	.26631	.37488	32.6	96	188	158	
Animation 4 (*Supplementary Movie 4, Figure S1.d*)										
Inducer Ring	70	**100**	80	.38911	.52548	32.6	153	183	12	
Cone Adaptation	69.704	279.34	60.501	.2754	.23594	32.264	188	157	235	
Cone Opponency	70	280	60.501	.27644	.23626	32.6	186	157	234	

B. Experiment 1: Fixed-Location Task (Figures S2-S3, Table S2-S3)

1. Colour Specifications and Results per Inducer (Exp 1)

Table S2. Colour specifications in Fixed-Location Task and Results per Inducer in *Experiment 1*. Dummy colours of the inducers are provided in the first column. The *CIE1931* chromaticity coordinates and luminance of the white-point were *xyY* = [0.3304, 0.3526, 101.1]. *Deviation from opponent*: statistics of t-tests across participants are reported comparing the difference of afterimage matches from cone-opponent predictions; *M* = average, *df* = degrees of freedom, *t* = t-value, *p* = probability of difference by chance *(*** p < .001, ** p < .01, * p < .05)*. The column *Naming* reports colour terms describing the inducers (*Indu*) and measured afterimage (*After*) colours: *R* = red, *O* = orange, *Y* = yellow, *G* = green, *T* = turquoise, *B* = blue, *Pu* = purple, *Pi* = Pink, *Br* = brown, *M* = magenta. Categories are indicated by the colour of the cells in the "Naming" column to illustrate transitions across hues. Hues close to the prototypes (within 10 deg) are highlighted in bold in the *Naming* column; for average measurements of prototypes and category boundaries, see *Table S3*.

	Inducer					Comp		Afterimage		Deviation from Opponent				Naming	
Nr	L*	Hue	chr	u*	v*	L*	chr	∅ Hue	Dummy	M	df	t	p	Indu	After
BG	70	-	0	0	0	-	-	-	171, 171, 170	-	-	-	-	Gy	
**EXPERIMENT 1a**															
1	58.1	7.2	235.7	233.9	29.5	58.1	40	198.5	101, 151, 159	11.3	30	5.3	***	**R**	B
2	74.9	27.6	133.8	118.6	61.9	74.9	35	226.6	160, 190, 217	19.0	30	10.0	***	**O**	B
3	98.1	87.9	101.2	3.8	101.1	65	40	270.3	157, 155, 203	2.4	30	2.0	.06	**Y**	Pu
4	86.6	142.5	177.8	-141.1	108.2	86.6	33	306.4	232, 208, 246	-16.1	30	-7.1	***	G	Pi
5	80.5	175.7	108.6	-108.3	8.0	80.5	30	356.7	223, 190, 197	1.0	30	0.6	.54	T	Pi
6	62.5	235.7	107.5	-60.6	-88.8	62.5	40	33.4	175, 143, 116	-22.3	30	-8.5	***	**B**	Br
7	42.8	289.1	141.0	46.0	-133.2	42.8	44	121.2	86, 111, 49	12.1	30	4.7	***	**Pu**	G
8	62.4	322.5	174.6	138.6	-106.2	62.4	65	148.8	99, 168, 114	6.3	30	4.0	***	M	G
**EXPERIMENT 1b**															
1	70	0	71	71	0	70	71	179.0	144, 180, 174	-1.0	50	-0.3	.73	Pi	B
2	70	15	71	68.6	18.4	70	71	201.4	144, 178, 187	6.4	49	2.2	*	**R**	B
3	70	30	71	61.5	35.5	70	71	222.4	150, 176, 197	12.4	49	5.0	***	O	**B**
4	70	45	71	50.2	50.2	70	71	240.3	156, 174, 203	15.3	50	6.6	***	**O**	B
5	70	60	71	35.5	61.5	70	71	247.6	160, 172, 204	7.6	49	3.1	**	O	B
6	70	75	71	18.4	68.6	70	71	263.4	167, 170, 206	8.4	50	4.6	***	**Y**	Pu
7	70	90	71	0	71	70	71	273.8	172, 168, 205	3.8	49	2.3	**	G	Pu
8	70	105	71	-18.4	68.6	70	71	284.0	176, 166, 204	-1.0	50	-0.5	.61	G	**Pu**
9	70	120	71	-35.5	61.5	70	71	296.2	181, 165, 200	-3.8	49	-1.6	.12	**G**	Pu
10	70	135	71	-50.2	50.2	70	71	315.6	188, 162, 193	0.6	51	0.2	.82	G	Pi
11	70	150	71	-61.5	35.5	70	71	330.2	191, 162, 185	0.2	51	0.1	.93	G	Pi
12	70	165	71	-68.6	18.4	70	71	349.5	193, 161, 173	4.5	50	2.0	.05	G	Pi
13	70	180	71	-71	0	70	71	3.5	194, 162, 164	3.5	51	1.5	.15	B	R
14	70	195	71	-68.6	-18.4	70	71	17.5	192, 163, 155	2.5	50	1.3	.22	B	O
15	70	210	71	-61.5	-35.5	70	71	25.9	191, 164, 150	-4.1	50	-2.0	.05	B	O
16	70	225	71	-50.2	-50.2	70	71	34.8	190, 165, 144	-10.2	50	-4.8	***	**B**	**O**
17	70	240	71	-35.5	-61.5	70	71	48.6	186, 167, 138	-11.4	50	-4.7	***	B	**O**
18	70	255	71	-18.4	-68.6	70	71	67.0	180, 170, 132	-8.0	50	-3.2	**	B	Y
19	70	270	71	0	-71	70	71	84.3	174, 172, 130	-5.7	50	-2.6	*	Pu	G
20	70	285	71	18.4	-68.6	70	71	107.5	165, 175, 134	2.5	51	1.2	.25	**Pu**	G
21	70	300	71	35.5	-61.5	70	71	123.3	158, 177, 140	3.3	50	1.5	.13	Pu	**G**
22	70	315	71	50.2	-50.2	70	71	132.8	155, 178, 145	-2.2	50	-1.1	.30	Pi	B
23	70	330	71	61.5	-35.5	70	71	146.3	150, 179, 153	-3.7	50	-1.5	.15	**Pi**	B
24	70	345	71	68.6	-18.4	70	71	155.6	147, 179, 159	-9.4	51	-3.7	***	Pi	B

2. Details on Afterimage Measurements (Exp 1)

Cover task. During the adaptation period (20s in *Exp. 1.a* and 30s in *Exp.1b*), a round fixation dot was blinking at a rate of 1Hz at the center of the screen. Observers were asked to stare at this dot. To make sure they would, they also completed a cover task. In some trials, the round dot would change for one blink (1s) into a square dot. Observers had to indicate at the very end of a trial, i.e., after matching the afterimage, whether such a square dot occurred in the trial or not. If their response to the cover task was wrong, they had to redo the whole trial at the end.

Intertrial Masking. During the intertrial period, a masking display canceled remaining afterimages to prevent interference across trials. For this, a display with moving colour circles was shown for 10 seconds. The display was based on *DotDemo.m* in *PsychToolBox*; 800 randomly coloured circles of different sizes move towards the centre of the screen and simultaneously, 800

colours moved from the centre away to the periphery. Observers were encouraged to move their eyes to counter afterimages. In the centre of the screen, a bar showed a countdown in steps of 1 second; the changes of the bar also stimulated eye movements. After the 10 seconds, a grey background with a fixation dot was shown and instructions asked observers to continue only after no colour is seen around the fixation dot anymore. Observers started the next trial by mouse click. Each trial began with a 1 second fixation dot to allow observers to get set up before the adaptation phase with the cover task started.

3. Control Task (Exp 1)

In this task, only the test display *(Figure 2.a)* was presented (i.e., there was no adaptation phase, cover task, or intertrial display). The display was the same as in the afterimage task, except for one difference: The centre disk was not grey but had the colour at the centre of the comparison range (i.e., the colour opponent to the inducer colour in the afterimage task). Observers were asked to choose the segment with the same colour as the one in the centre. The response mode was the same as in the afterimage task, including the limitation of response time to 5 seconds.

Results are shown in *Figure S1*. Contrary to the main task in *Figure S2.a*, the matches in the control task *(Figure S2.b)* lie exactly on the hue directions of the cone-opponent comparison stimuli. The results of the control task show that the biases in colour selection in the afterimage task can neither be attributed to technical artefacts, such as imperfections of device calibration, nor to nonlinearities in stimulus sampling and representation, nor to unspecific response biases, for example because of the variation of discriminability between the comparison colours *(cf. Witzel & Gegenfurtner, 2018; Witzel et al., 2019)*.

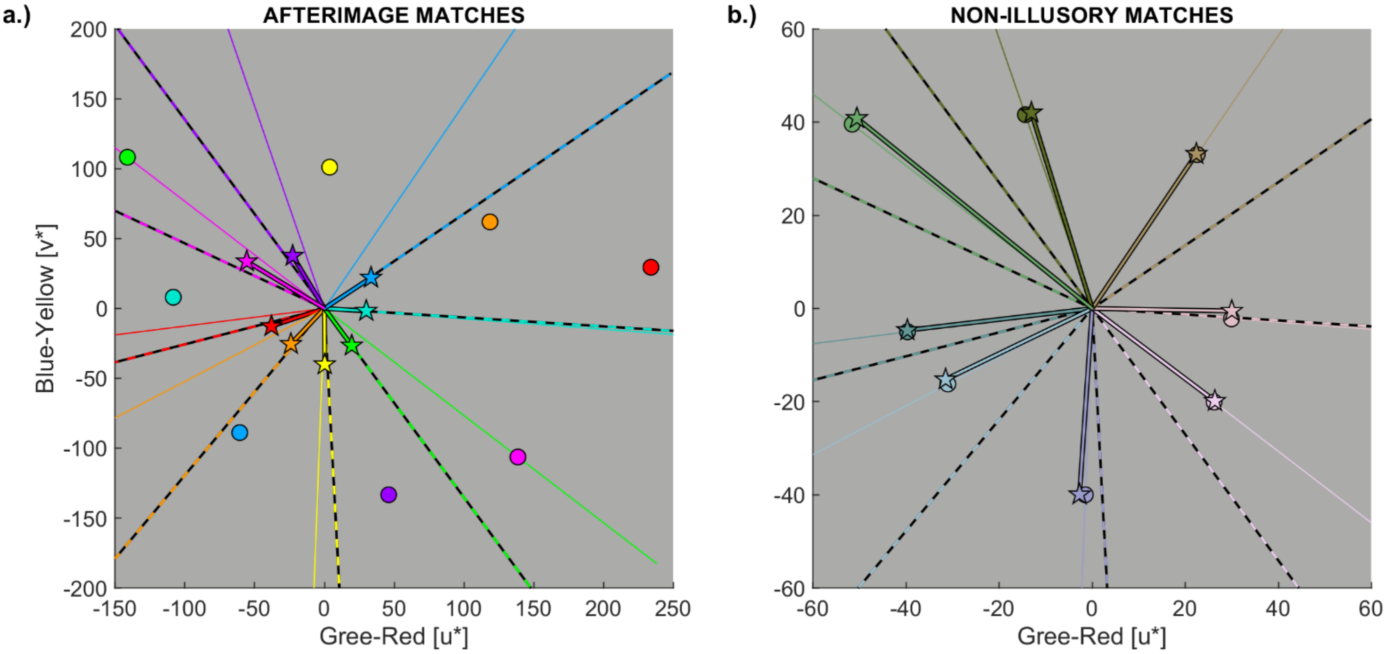

Figure S2 | Afterimage Matches & Control Task in *Experiment 1a*. Panel a shows the afterimage matches like *Figure 2.b*, but for all eight inducer colours instead of only the four examples. For comparison, panel a illustrates the non-illusory matches of real colours in the control task. Format as in *Figure 2.b* except for the following simplification. The stimulus colours are shown as circles only (i.e., without the bold lines as in *Figure 2.b*) to avoid clutter; the stimulus colours are the inducers in panel a, and the matching targets in panel b. Note: The afterimage matches (stars in panel a) are in line, or at least very close to, the prediction by cone adaptation (dashed line); in contrast, the non-illusory matches (stars in panel b) are all in line, or very close to, the target colour (circle underneath the stars) rather than cone adaptation (dashed lines). The results on non-illusory colours in panel b show that the results for afterimages in panel a cannot be explained by a stimulus bias, a technical glitch, or response biases in hue discrimination.

4. Colour Naming (Exp 1)

Colour categories are the ensembles of different colours that can be described by a colour term; for example, all the different types of greenish colours that are all called green. Prototypes are the best examples of colour categories, for example the green that is greener than any other member of the green category. In contrast to prototypes, unique hues are the red, yellow, green, and blue hues that appear to be pure and unmixed. Unique hues correspond to Hering colours. These are not necessarily the same as the typical hues of the red, yellow, green, and blue categories, but they are nevertheless very similar (*Witzel & Gegenfurtner, 2018; Witzel et al., 2019)*.

Experiment 1a. The prototypes of colour categories were measured in separate blocks from the afterimage measurements in *Experiment 1.a*. Participants were shown the circle with the comparison colours and were asked to choose the one that is closest to the typical colour of a colour term. They were only asked for the prototype of one colour term in each set of comparison colours. Those colour terms were chosen to best correspond with each set of comparison colours. For the comparisons of the red, orange, yellow, green, cyan,

blue, purple, and magenta inducers, they were asked for typical *blue*, *blue*, *purple*, *pink*, *pink*, *brown*, *green*, and *green*, respectively (cf. last column in *Table S2*).

Experiment 1.b. After the main part of *Experiment 1.b*, colour categories and prototypes were measured following previous procedures *(Witzel & Gegenfurtner 2018; Witzel et al., 2019)*. The 24 isoluminant colours used as inducers in the afterimage task *(Table S2)* were presented in random order as coloured disks at the centre of the otherwise grey screen. Observers named these colours with the eight chromatic basic colour terms by pressing one of eight corresponding keys. The German chromatic basic colour terms are the equivalents of pink (*rosa*), red (*rot*), orange (*orange*), yellow (*gelb*), green (*grün*), blue (*blau*), purple (*lila*), and brown (*braun*). After the categorisation task, observers adjusted the hue of a randomly coloured disk to match the typical hue of a colour term. The adjustable colour was sampled from the isoluminant hue circle and presented at the centre of the screen. In each trial, a written colour word indicated which prototype to adjust. A block with naming all 24 colours and adjusting the prototypes was repeated three times. 50 of the overall 51 participants of *Experiment 1.b* took part in the colour naming measurements.

Results & Discussion. Colour categories depend on hue, lightness and chroma. Despite the differences in stimuli and procedure, the most typical hues are similar between *Experiment 1.a* and *Experiment 1.b*. The largest differences occur for green and purple. The constant lightness and the low chroma in *Experiment 1.b* may explain some of the variation of typical hue adjustments. In particular, there is no hope to obtain a proper example of red in that desaturated stimulus set. Nevertheless, these adjustments are in line with previous measurements of typical and unique hues at typical lightness levels of the corresponding colour categories *(Figure 1 in Witzel & Gegenfurtner, 2018; Figure 1-2 in Witzel et al., 2019)*.

The key result reported in the main article is that colour naming data contradicted the description of afterimage colours in terms of Hering opponency. In both versions of *Experiment 1*, naming of inducers and afterimages does not correspond with Hering colours. *Figure S3* illustrates this for *Experiment 1.a*. The afterimage of saturated red is close to typical blue, the afterimage of yellow is almost exactly at typical purple, the one for bright green is pink, and the one for blue is brown. Conversely, an almost perfectly blue afterimage is produced by orange instead of yellow, and green afterimages are produced by purple and magenta, not red.

Results from *Experiment 1.b* are similar: Red inducers produced "blue" (instead of green) afterimages. Yellow induced purple (instead of blue). Green induced purple or pink (instead of red), and a green afterimage is produced by a purple inducer. Results for blue inducers are more variable because blue inducers produce faint, desaturated afterimages *(cf. Figure 2.f)* that are often called brown (see *Experiment 1.a* above, or *Figure S3)*. Typical brown is closer to orange than to yellow in both measurements *(Table S3)*, implying that the afterimage of blue has an orangish hue, contrary to the yellow predicted by Hering opponency.

Table S3 | Results from Colour Naming in *Experiment 1*. The operator ± indicates standard error of mean. Dummy Colours for average prototypes assuming sRGB monitors are provided for roughly approximate illustration in the "Dummy" column; note that they may look slightly different on a calibrated display. For *Experiment 1.a* (top), the average prototypes choices are given for the comparison colours corresponding to the red, orange, yellow, green, turquoise, blue, purple, and magenta inducers, respectively. In *Experiment 1.a*, lightness (L*) and chroma varied and are provided for reference. For *Experiment 1.b*, Lower and Upper refer to lower and upper boundary in the categorisation task. The upper boundary of one term is the lower boundary of the subsequent term. For comparison, the columns under *Previous* report results at typical lightness levels from previous studies *(Witzel & Gegenfurtner, 2018; Witzel et al., 2019).*

Experiment 1a					Previous		
Term	*Prototype*	*Lightness*	*Chroma*	*Dummy*	*Typical*	*Unique*	*L**
Blue	223.3 ± 1.0	58.1	40	81,148,173	227.4	224.9	60
Blue	228.5 ± 1.8	74.9	35	145,191,216			
Purple	273.8 ± 1.7	65	40	162,151,201			
Pink	329.3 ± 1.7	86.6	33	254,202,233			
Pink	331.1 ± 2.2	80.5	30	234,186,213			
Brown	52.8 ± 1.6	62.5	40	179,146,106			
Green	132.0 ± 2.2	42.8	44	48,114,60	130.7	136.9	50
Green	131.1 ± 2.2	62.4	65	75,170,90			

Experiment 1b					Previous		
Term	*Prototype*	*Lower*	*Upper*	*Dummy*	*Typical*	*Unique*	*L**
Pink	335.2 ± 2.1	302.9 ± 1.7	2.8 ± 2.3	206,158,182			
Red	10.6 ± 1.7	2.8 ± 2.3	15.3 ± 1.4	207,160,161	17.5	7.9	50
Orange	42.3 ± 1.1	15.3 ± 1.4	58.7 ± 1.4	198,165,143			
Brown	52.8 ± 2.6	58.7 ± 1.4	60.2+1.6	193,167,139			
Yellow	72.2 ± 1.4	60.2+1.6	85.1 ± 1.4	182,171,133	77.8	79.7	76
Green	125.1 ± 1.8	85.1 ± 1.4	176.3 ± 1.1	146,180,143	130.7	136.9	50
Blue	227.9 ± 1.8	176.3 ± 1.1	257.7 ± 1.0	138,177,198	227.4	224.9	60
Purple	283.0 ± 1.3	257.7 ± 1.0	302.9 ± 1.7	181,164,203			

5. Additional Results from Experiment 1.a

By showing the results for all inducers, *Figure S2.a* complements *Figure 2.b* in the main article. However, results for all inducers are better appreciated by loking at each inducer separately in *Figure S3*. The key result reported in the main article is that the average response (vertical black line) deviates systematically from the cone-opponent hue (bar with black edge) and is close to the prediction through cone-adaptation (dashed red line) in all panels. In addition, the prototypes close to the afterimages do not correspond to the colour terms according to Hering opponency (cf. *Results & Discussion* in *A.4 Colour Naming).*

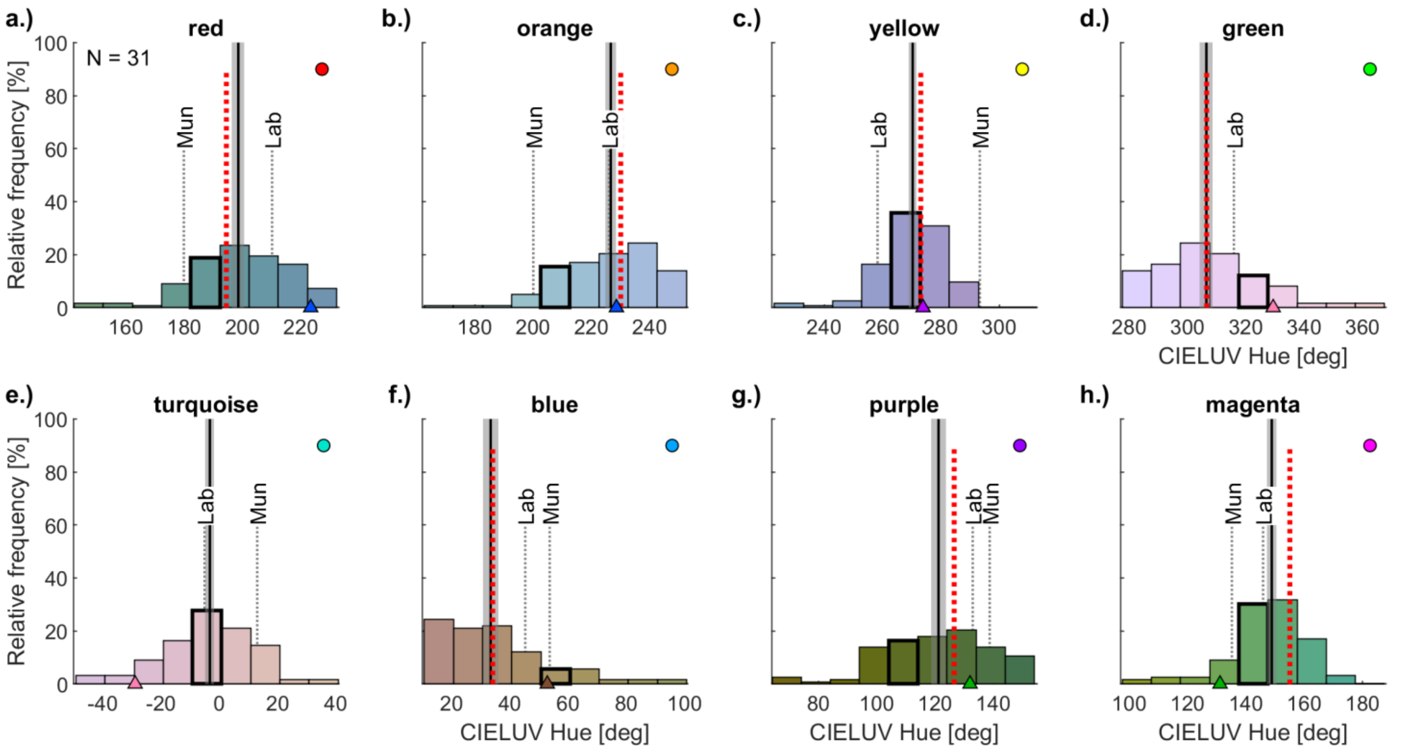

Figure S3 | Hue Histograms for Experiment 1a. Results for each inducer are shown in separate panels. The inducer colour is described by the title and reproduced by the coloured circle in the top right corner. In each panel, the bars correspond to the 9 comparison colours observers chose from (see Method in main article). Accordingly, the x-axis represents hue in intervals of 10-degree azimuth in CIELUV; the height of the bars shows how often each comparison colour had been chosen across the 124 trials (4 repetitions *31 participants). The bar highlighted by the thick black edge corresponds to the cone-opponent colour (same in DKL and CIELUV). The black vertical line with the grey shade indicates the average and the standard error across individuals, where individual data was aggregated by median across blocks. The dashed red line indicates the prediction through cone adaptation. The thin, dotted vertical lines correspond to the predictions by opponency in CIELAB (*Lab*) and the Munsell system (*Mun*). In panel d, the Munsell prediction (19.6 deg) is outside the displayed x-axis range. The triangles along the x-axis indicate the prototypes chosen among the comparison colours. The colours of the triangles indicate the respective colour term: blue, blue, purple, pink, pink, brown, green, and green for each panel, respectively.

C. Experiment 2: Chaser-Like Task (Figures S4-S12, Table S4)

1. Details on Participants and Apparatus (Exp 2)

Table S4 | Participant & Monitor specifications in *Experiment 2*. CIE1931 Measured chromaticities (x, y), luminance (Y) in *cd/m²*, and gamma (γ) are given for the red (*R*), green (*G*), and blue (*B*) primary and for white (*W*). Index ^{m} refers to measured and ^{c} to computed values. *BG* reports computed chromaticities and luminance of background. *Chroma* refers to the specified inducer chroma. It was set so that inducers and extreme adjustments of afterimages are within the respective monitor gamut. Participants, with whom the respective monitor was used, are provided in the last row. The ID indicates their biological sex (*f* = female, *m* = male) and ages in years are given in parentheses *(cf. Figure S8-S10)*; *cw* = the author.

	**Monitor 1**				**Monitor 2**				**Monitor 3**			
	x	y	Y	γ	x	y	Y	γ	x	y	Y	γ
**Experiment 2a CIELUV**												
R^m	.6467	.3354	42.5	**2.31**	.5801	.352	53.1	**2.03**	.6851	.3124	87.2	2.25
G^m	.3130	.6017	190.4	2.32	.3287	.5436	166.8	2.03	.2157	.7223	237.5	2.22
B^m	.1480	.0504	17.0	2.39	.1533	.1197	41.4	2.05	.1504	.0444	19.0	2.23
W^m	.2956	.3197	252.0	2.25	.3004	.3252	262.2	1.99	.3134	.3299	339.5	2.19

**BG^c^**	.2957	.3200	101.8	-	.3004	.3251	106.5	-	.3151	.3316	140.1	-
**Chroma**	42				38				50			
**Participants**	*cw* (41y), *f1* (38y)				*f2* (19y)				*f3* (20y), *f4* (19y), *f5* (19y), *f6* (19y), *m2* (19y), *m3* (20y), *m4* (20y)			
**Experiment 2b DKL**												
**R^m^**	.6493	.3417	44.1	2.49								
**G^m^**	.3232	.6156	168.1	2.51								
**B^m^**	.1484	.0581	25.2	2.54								
**W^m^**	.284	.2858	237.1	2.51								
**BG^c^**	.2828	.2838	118.7	-								
**Chroma**	0.5 (*cw*) and 0.7 (*f1*)											
**Participants**	*cw* (45y), *f1* (42y)											

2. Data Distributions (Exp 2)

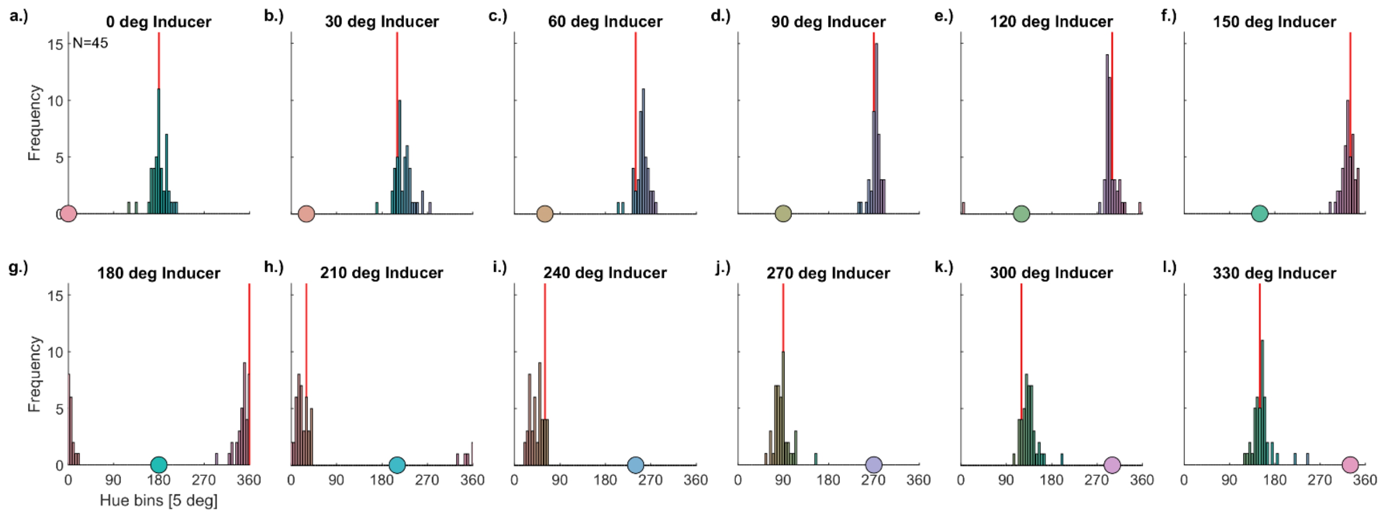

Figure S4 | Example Distributions of Afterimage Hue Measurements in *Experiment 2a*. Each panel shows a histogram of the adjusted afterimage hue for one of 12 example inducers. The x-axis corresponds to the hue bins, the y-axis to frequency of each bin. The examples are inducer hues at 0, 30, 60, 90, 120, 150, 180, 210, 240, 270, 300, and 330 degrees azimuth. The inducer colour is illustrated by the coloured disk; the colours of the histogram bars visualise the respective afterimage colours. All 45 measurements are pooled from the repeated measurements of the 10 participants. Bins are 5 degrees wide. The red line indicates the cone-opponent hue (DKL, CIELUV). Note the roughly normal, symmetrical shape of the distributions, and the deviations of the central tendencies from the cone-opponent hue for some inducers (e.g., 60 deg, 240 deg, 300 deg). Single outliers may come from some naïve observers seeing very faint afterimages.

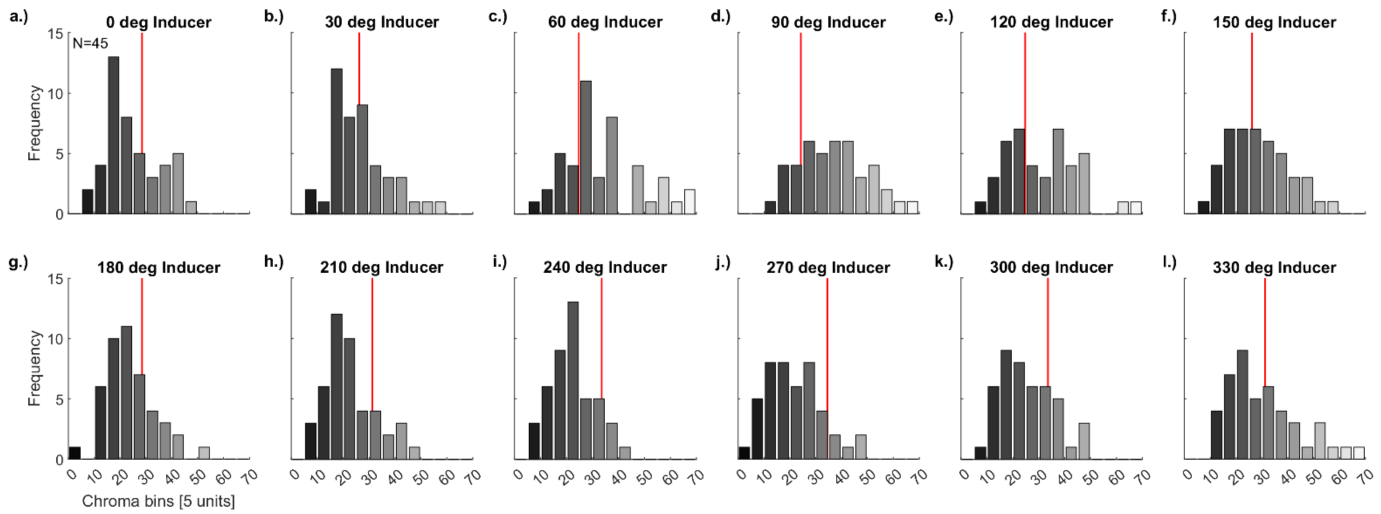

Figure S5 | Example Distribution of Afterimage Chroma Measurements in *Experiment 2a*. Format is as in *Figure S4*, except that bin width is 5 units in CIELUV chroma. The cone-opponent prediction (red line) corresponds to the prediction in DKL-, but not CIELUV-space, as the two spaces differ in terms of predicted chroma. Note again the roughly normal distribution despite the fact that inducer chroma varied across participants (see *Table S4*). A few measurements are within the 0-5 bin (leftmost bar in panels *g* & *j*). This means that in those trials the participant adjusted the colour to grey, implying that no afterimage has been seen. Also note that the central tendency is clearly below the cone-opponent prediction for some inducers (e.g., 210-330 deg in panels *h-l*), but above the cone-opponent prediction for other inducers (60-120 deg in panels *c-e*). These

observations cannot be explained by a simple under- or overestimation (uniform shift in one direction). They thus further highlight the difference of afterimages from cone-opponency.

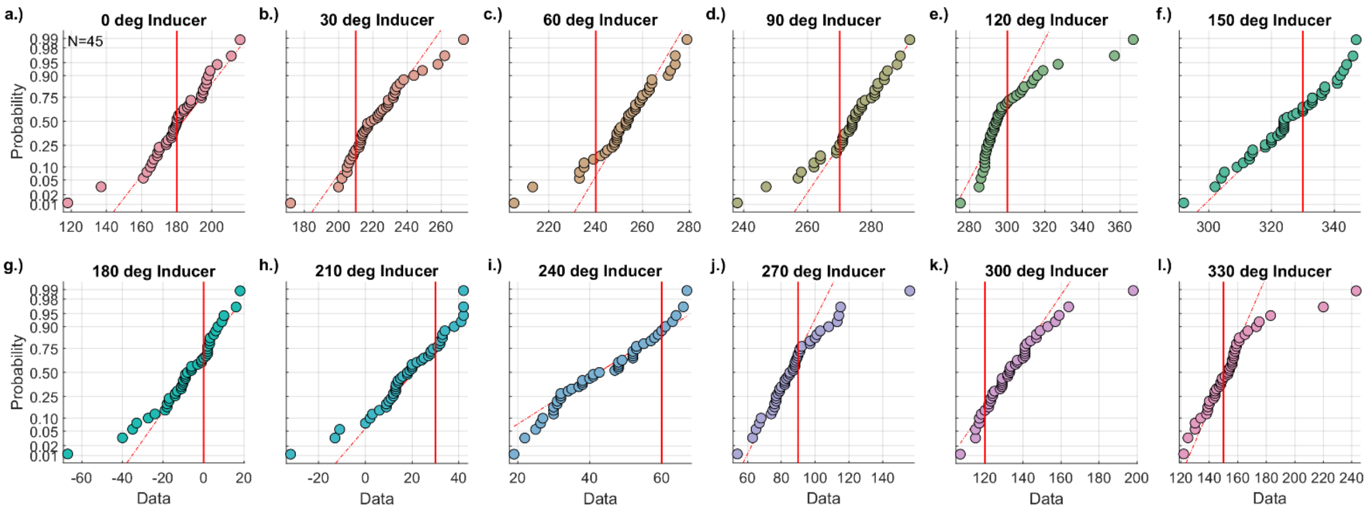

Figure S6 | Normal Probability Plot for Afterimage Hue Measurements in *Experiment 2a*. The x-axis indicates measured hue angle of afterimages; the y-axis corresponds to the probability of normally distributed data. The coloured circles refer to single hue measurements. The dashed red line indicates perfect normal distribution. Deviations from the red line suggest non-normal data variation. For most inducers, most of the data fall onto the line. There are some obvious outliers at the extreme ends of the distribution for 300 degree and 330 degrees (panels *k-l*). For inducers of 60-120 degrees (panels *c-e*), the deviations seem to be more systematic. The reason for these is likely the variation of inducer strength across participants, which has an effect on perceived hue, as shown in *Experiment 3*.

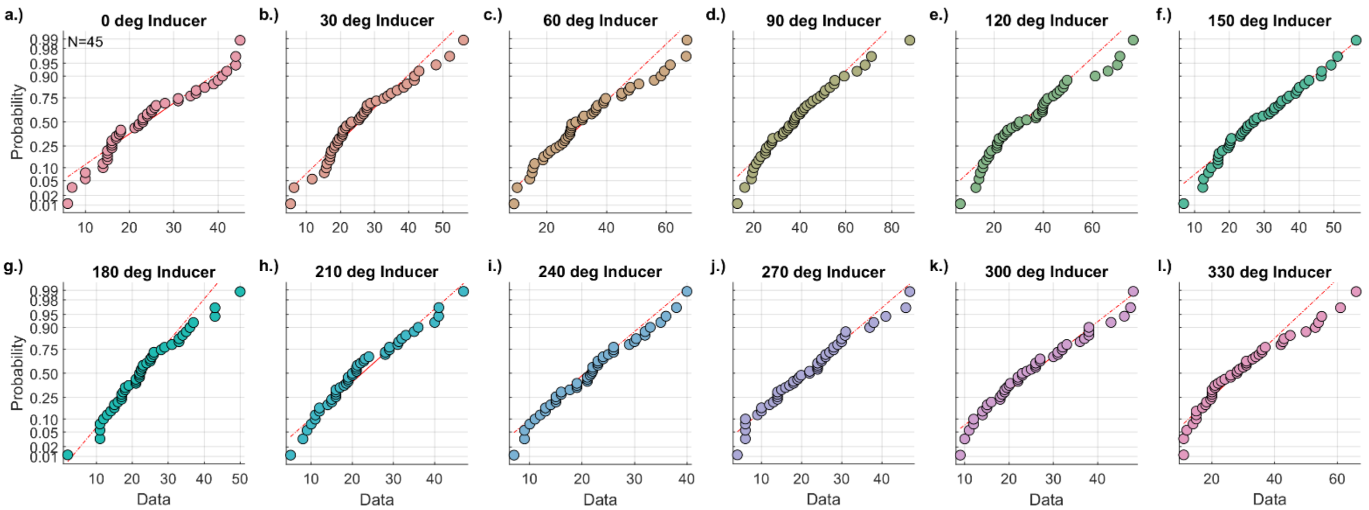

Figure S7 | Normal Probability Plot for Afterimage Chroma Measurements in *Experiment 2a*. The format is as in *Figure S6*. The data is slightly positively skewed by the mere fact that there is no negative chroma, and that afterimages, by experimental design, were not strong enough to be limited by the monitor gamut. The deviations affect only very few extreme data points. They are most pronounced for 60-90 degree (panels *c-d*), in line with the observation that hue was slightly skewed for those inducers, and in line with the relationship between afterimage chroma and hue shown in *Experiment 3*.

3. Experiment 2.a: Results on Individual Observers

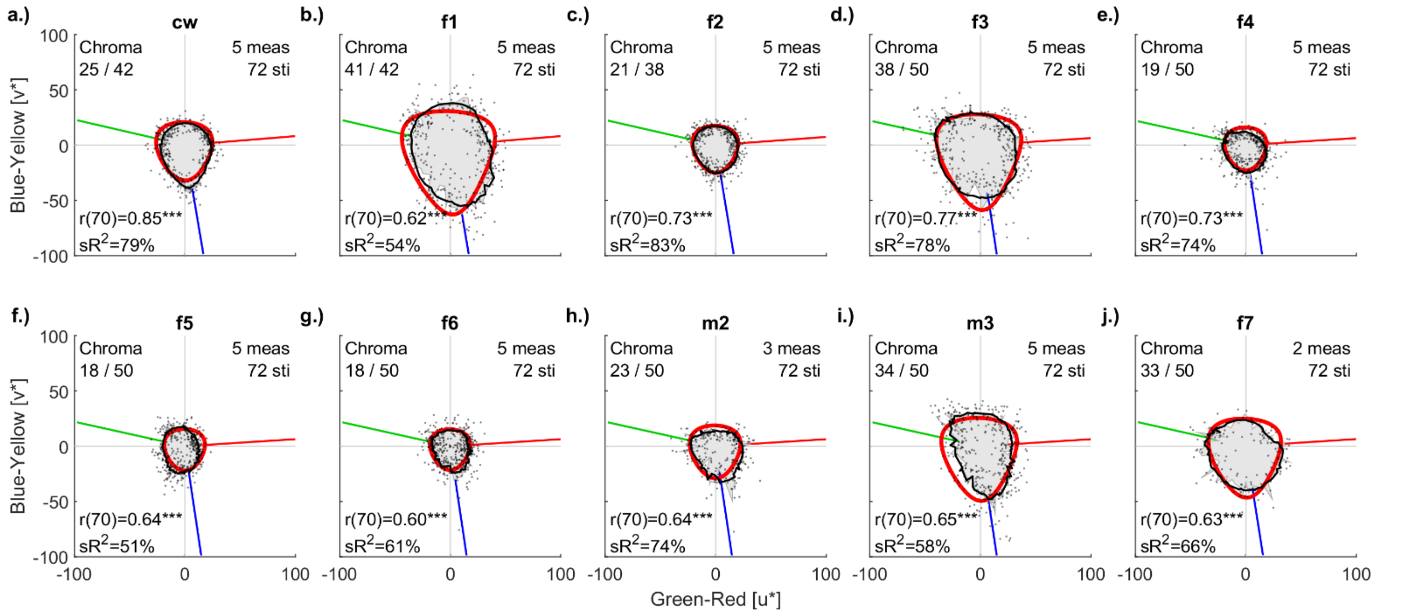

Figure S8 | Averages for Individual Observers in *Experiment 2a*. The black line illustrates the average adjustments of each observer. Averages are calculated across repeated measurements. The dots correspond to the single data. The red curves are the afterimages predicted by the cone-adaptation model. *Chroma*: The first value is the rounded grand average chroma of measured afterimages, assumed to reflect the strength of afterimages in the simulations; for comparison, the second value indicates inducer chroma (cf. *Table S4*). *Sti* = Number of inducers, *meas* = number of repeated measurements; the correlations in the bottom left corner are between simulated and measured chroma, and *sR²* is the percentage of variance explained by the smoothed data (moving average across 9 neighbouring data), provided as a rough indicator of model fit in the absence of measurement noise. Note that all correlations are significant confirming the results with aggregated data in the main article *(Figure 2.f)*.

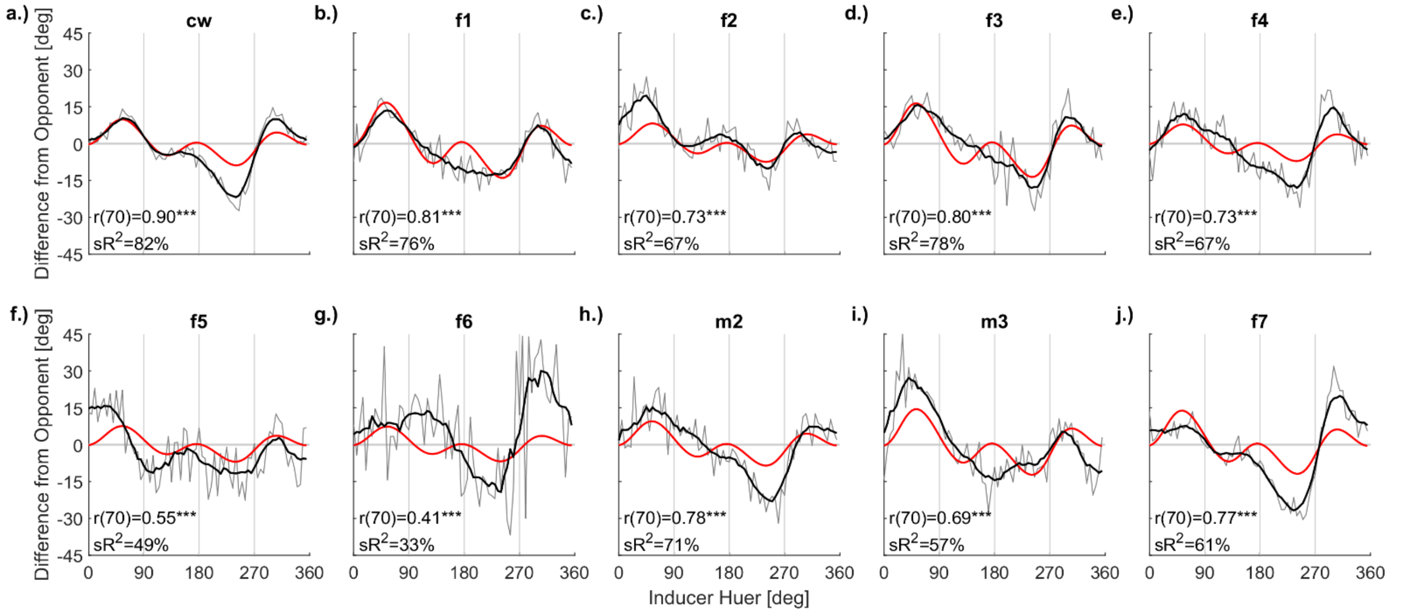

Figure S9 | Deviations from Cone-Opponency for Individual Observers in *Experiment 2a*. Deviations from cone-opponency are differences between the hue opponent to the inducer (inducer hue-180 deg) and the hue of the afterimage predicted by the cone-adaptation model *(red line)* or the hue of the measured afterimage *(grey line)*. Averages are calculated across repeated measurements. The black line shows the data smoothed by moving average across nine neighbouring data. The correlation in the bottom left corner is between simulated and measured deviations from opponency, and *sR²* is the variance explained by the smoothed data. Note that all correlations are significant, confirming the main results with aggregated data (*Figure 2.g*) at the individual level.

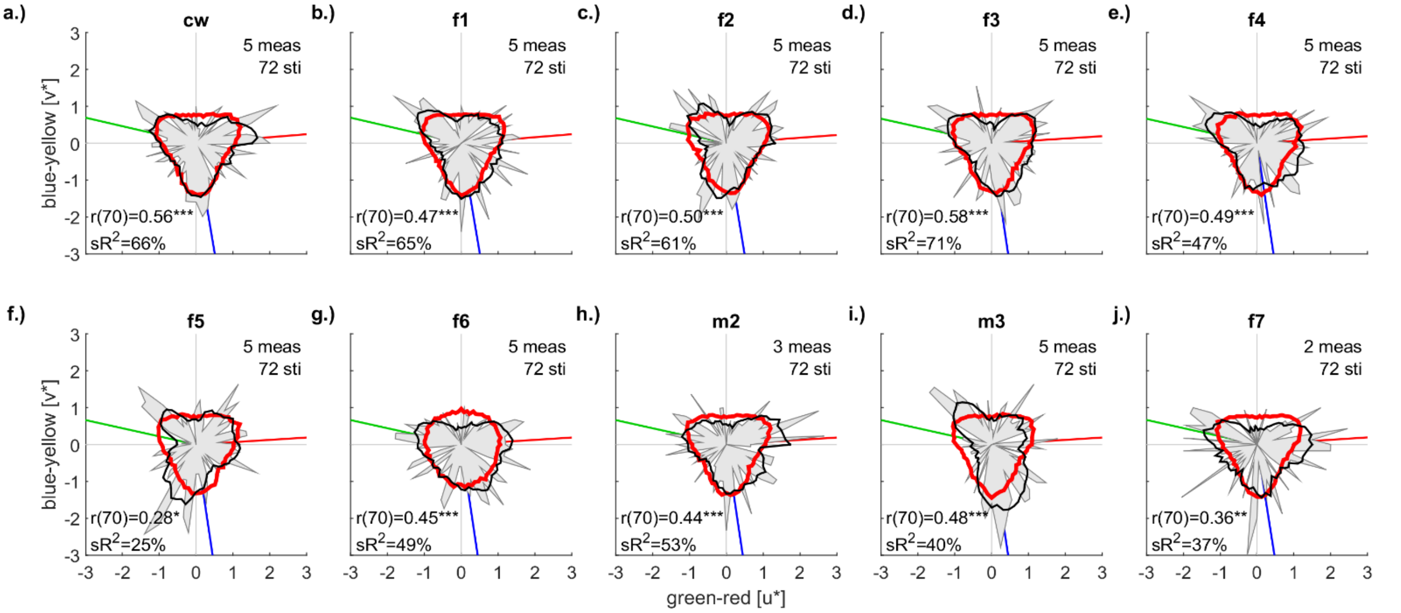

Figure S10 | Polar Hue Histogram for Individual Observers in *Experiment 2a*. Each histogram is calculated for the *N* hues * *k* repetitions indicated at the top right of the diagrams. The correlation in the bottom left corner is between predicted and measured histograms, sR^2 for the explained variance of smoothed data. Note that all correlations are significant confirming the results of the main article (*Figure 2.h*) with aggregated data.

4. Experiment 2.b: Sampling in DKL Space

Figure S11 shows that sampling in DKL space (*Figure 11.a)* is expected to produce even stronger afterimages in the purplish S-direction *(Figure S11.b)* than sampling in CIELUV space *(Figure 1.b)*. In DKL, the afterimages in the *S*-direction are so strong that the grand average chroma is almost the same as the inducer chroma (0.47 vs 0.5) for participant *CW* and even higher than inducer chroma for participant *f1* (0.71 vs 0.7). This complicates stimulus sampling in DKL-space in two ways:

(1) The stronger shift of afterimages in the purplish direction dominates the predictions through cone adaptation. As a result, the *S*-pole dominated pattern of cone-adaptation in DKL space is less characteristic than the more distinctively tripolar pattern obtained in CIELUV space (cf. *Figure 1.b*).

(2) Chroma had to be lowered in *Experiment 2.b* to avoid measurements reaching the display gamut. However, the difference between the afterimage strength along the S-cone direction and the comparative weakness of afterimages in the other hue directions made such a sampling almost impossible in DKL-space. For this reason, the chosen chroma setting in *Experiment 2.b* is a compromise between avoiding the gamut and keeping saturation along the circle sufficiently high to obtain clear afterimages in hue directions other than the purplish *S*-cone direction. At close inspection, some limitations of this approach are visible in *Figure S12*. A few individual measurements (grey dots) reach the gamut in the S-cone direction, especially for observer *f1*, indicating that the gamut limited the saturation of the adjustment. This clipping effect of the display gamut along the S-cone direction undermines the match of measurements and predictions of chroma (*Figure 12.a,d,g*).

The detrimental effects of the gamut were obviously insufficient to break the fit between predictions and measurements, as the measurements for the DKL-sampled stimuli were all clearly correlated with the cone-adaptation model (*Figure 12*). Nevertheless, the sampling in CIELUV allowed for a better stimulus control and cleaner measurements of afterimage colours. It has therefore been the main approach to stimulus sampling adopted in this study (see *Method*).

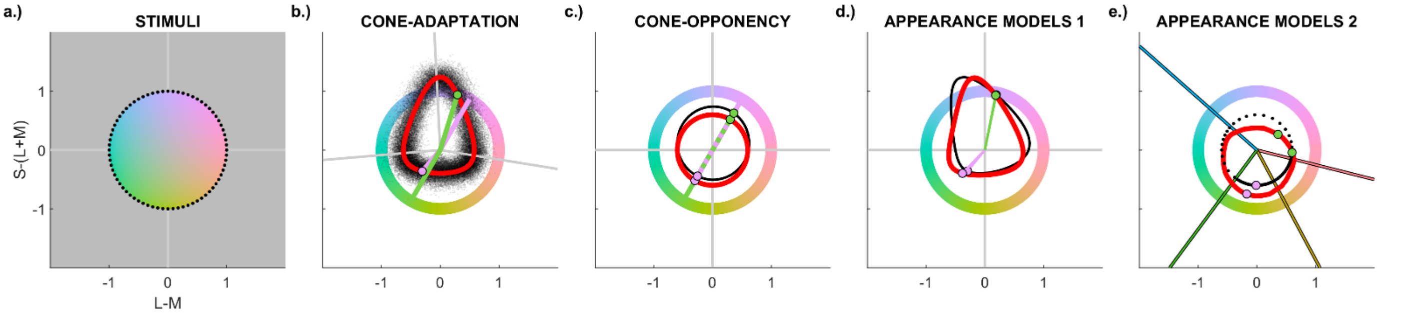

Figure S11 | Afterimage Stimuli and Models in DKL-Space. The figure is analogous to *Figure 1* in the main article, with the x-axes corresponding to the cone-opponent *L-M* channel and the y-axes to the S-*(L+M)* channel. Panel *a* illustrates the 72 hues that served as stimuli in *Experiment 2b*. Note the highly saturated afterimages in the *S*-direction predicted by the cone-adaptation model in panel *b*.

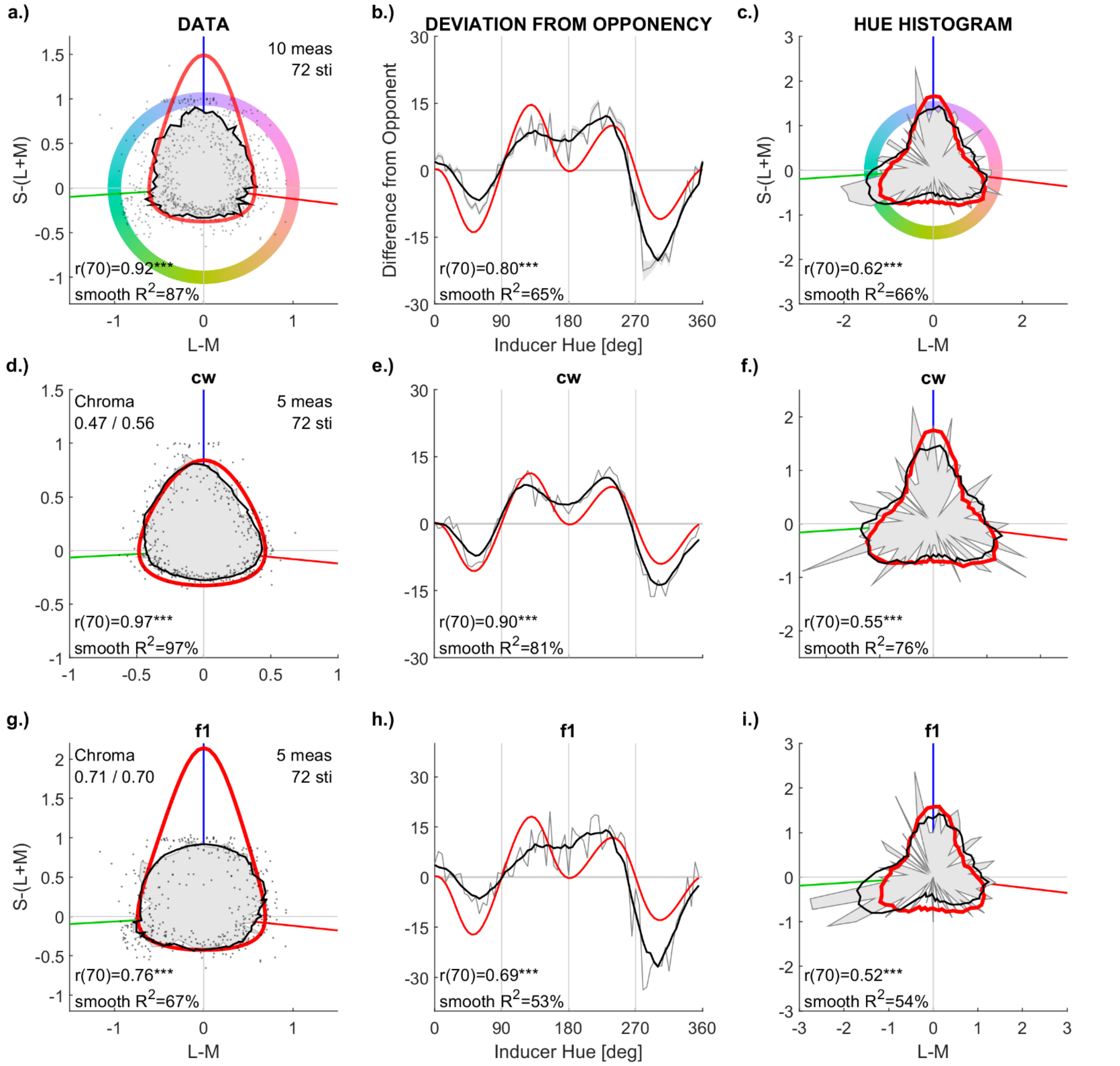

Figure S12 | Results for *Experiment 2b*. Unlike *Experiment 2a*, inducer colours in *Experiment 2b* were sampled along a circle in DKL- instead of CIELUV colour space. Five repeated measurements with N = 72 colours were done for two observers (*cw*, *f1*), everything else was as in *Experiment 2.a*. The first row illustrates results aggregated across the two observers comparable to *Figure 2.f-h* in the main article. The second and third row show individual results comparable to *Figures S8-S10*. All results confirm those from *Experiment 2a*.

D. Experiment 3: Effects of Inducer Chroma on Afterimage Hue (Figure S13, Table S5)

1. Details on Participants and Apparatus (Exp 3)

Table S5 provides details on *Participants* and *Apparatus* in *Experiment 3*. Apart from the author, CW, the other four participants did not know the purpose of the experiment. *CW* and *f7* had also participated in *Experiment 2*; the other three were exclusively recruited for *Experiment 3* and did not have any prior experience with the task.

Table S5 | Participant, Monitor, & Stimulus specifications in *Experiment 3*. Each row corresponds to the participant indicated in the first column *Participant*. The symbol * identifies participants who also took part in *Experiment 2*. *Monitor* numbers refer to those specified in *Table S4*. *Chroma levels* report the different levels of chroma tested for the respective participant; *max* refers to the maximum chroma possible. The range of the settings for maximum chroma across hue are provided in parenthesis. As the chroma of 50 was close to the monitor gamut for a few hues, those hues were set to a chroma of 35 in the *maximum-chroma* condition to avoid spurious gamut effects on adjustments and still get an additional datapoint. This is why the lowest value of the range in parentheses is smaller than 50. *Hue* indicates the number of hues and, in parentheses, the difference between adjacent stimulus hues, for which afterimages were measured in *Experiment 3*. *Rep* gives the number of repetitions of the stimulus conditions (these numbers varied depending on participant availability). *CW* had completed 5 repetitions at chroma 42 as part of

Experiment 2, but did only 2 repetitions at chroma 20, and only 1 at chroma 30 and maximum chroma. Participant *f8* completed only measurements with 8 hues (45 deg differences) at chroma 20. *Dataset* provides the size of each dataset after averaging across repeated measurements; these sizes are the combinations of chroma levels (2, 3, or 4) and hues (8, 24 or 72) and correspond to those reported in *Figure 3* and *Figure S13*. Column *Figure* indicates where to find the diagrams with the results for that participant.

Participant	*Sex*	*Age*	*Monitor*	*Chroma levels*	*Hue*	*Rep*	*Dataset*	*Figure*
CW*	m	43	1	20, 30, 42, max (31-102)	24 (15 deg)	1, 2 or 5	4*24 = **96**	S13.a-d
f7*	f	19	3	20, 50, max (35-120)	72 (5 deg)	2-3	3*72 = **216**	3
f8	f	19	3	20, 50, max (35-120)	24 (15 deg)	1 or 2	8+2*24 = **56**	S13.e-h
f9	f	19	3	50, max (35-120)	24 (15 deg)	4-5	2*24 = **48**	S13.i-l
f10	f	19	3	20, 50, max (35-120)	24 (15 deg)	2-5	3*24 = **72**	S13.m-p

2. Additional Results on the Effect of Inducer Chroma on Afterimage Hue (Exp 3)

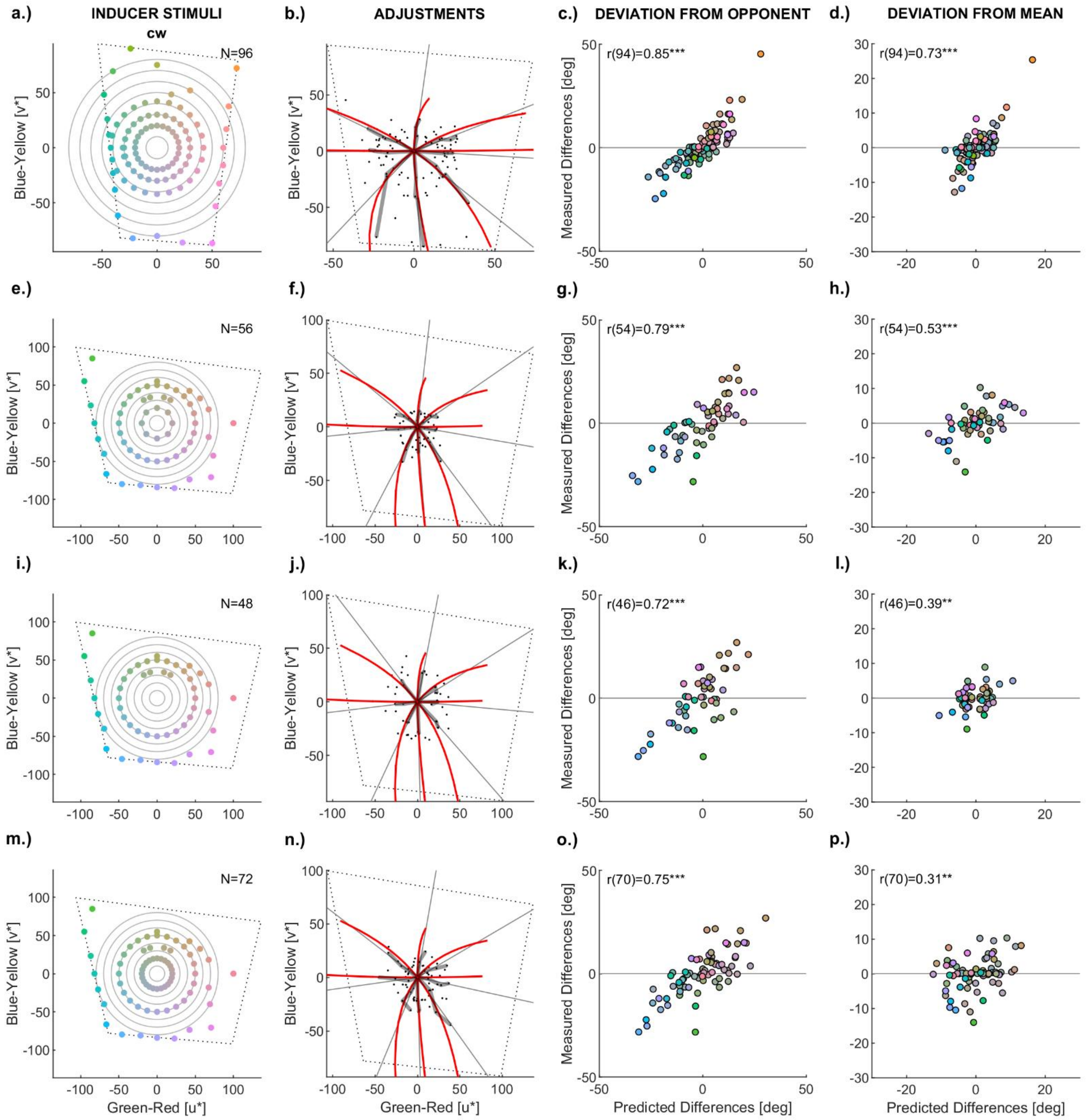

Figure S13 | Additional Data on the Effect of Inducer Chroma on the Hue of Afterimages (*Experiment 3*). Format is as in *Figure 3* of the main article. The only difference is that these data are from four other observers, which are indicated on top of the images in the first column *(panels a, e, i, & m)*. All observers show the predicted correlations, replicating the results for observer *f7* in the main article.

E. Models (Figure S14)

1. Cone-Contrast Model

Code S1 provides the Matlab code that implements cone adaptation as presented in the main article and formalised there in *Equation 1*. *Figure 14* illustrates the colour properties that determine the nonlinear change of hue when inducer hue and chroma vary (as tested in *Experiments 1-3*).

The comparison between first and second row of *Figure S14* shows that afterimages are dominated by the cone that is least adapted in comparison to the other two cones *(second row)*, not by high adaptation of single cones *(first row)*. This may appear confusing but follows from the fact that the strongest change in the multiplicative inverse happens at low cone excitation and flattens towards higher cone excitation *(Figure S14.a & d)*. So, low levels of adaptation dominate the induced colours, not high levels of adaptation.

The lines in *Figure S14.e-f* correspond to the isolated-cone excitation lines in *Figure 1.b*, *Figure 2.d,f,h*, and *Figure 3.c*. These isolated-cone lines are unrealistic and only serve the purpose of illustrating the extreme case to which adaptation converges with increasing saturation (inducer strength). Because cones cannot be stimulated in an isolated fashion (particularly *L*- and M-cones), peaks of adaptation can never be perfectly aligned with those lines.

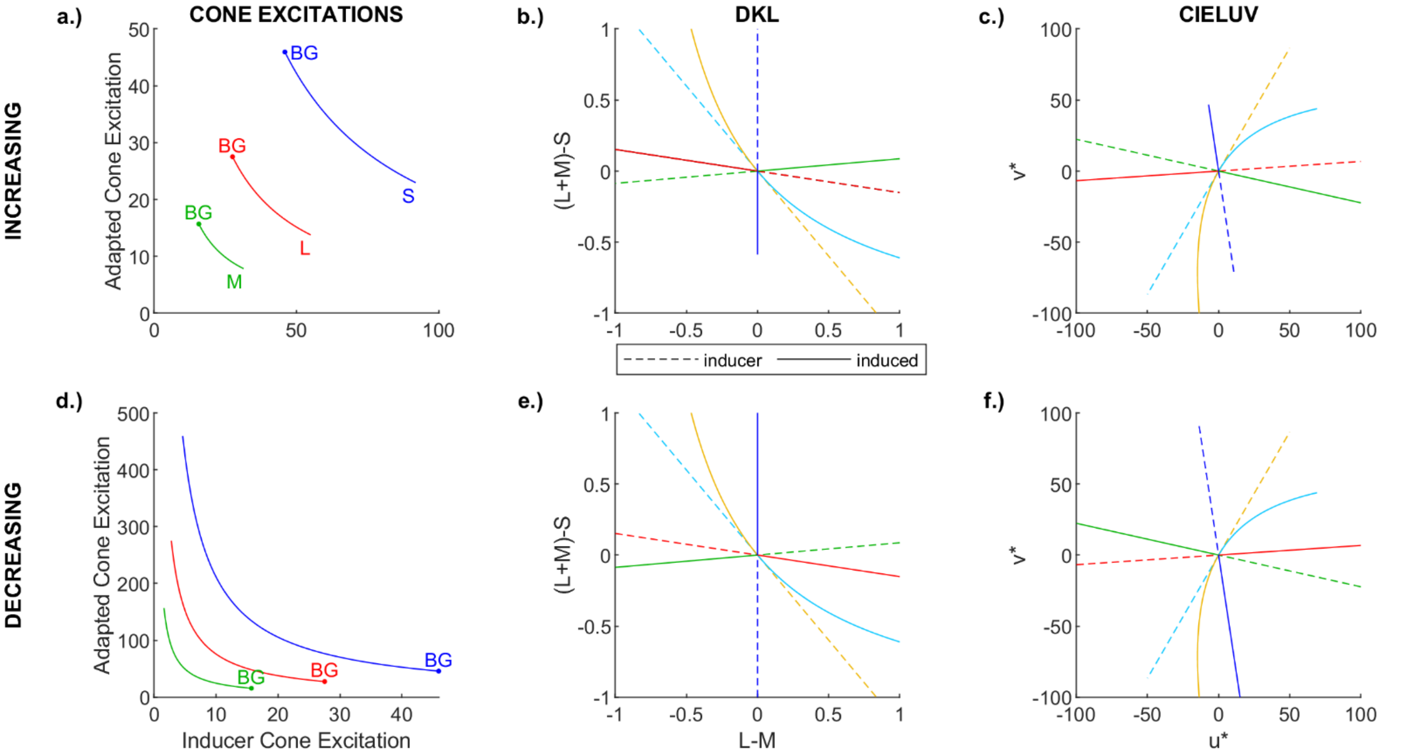

Figure S14 | Determinants of Non-Linearities. The first and the second row illustrate the extreme case of increasing and decreasing adaptation of a single cone when adaptation of the other cones is held constant. Increasing adaptation means that the excitation of one cone by the inducer increases starting from the excitation level of the background (from left to right along the x- axis in *panel a*). Decreasing adaptation means that the excitation of the cone by the inducer decreases starting from the background excitation level (from right to left along the x-axis in *panel d*). In both cases, the inducer excitation of the respective other cones is held constant at background excitation level *(dots with the label BG in panels a & d)*. Each of the three coloured curves illustrates this for changing one of the three cone types (red curve for changing *L*-cone excitation, green for *M*-cone, and blue for *S*-cone excitation). The y-axes in panels *a* and *d* show the excitation of the adapted cone when responding to a constant, neutral background (*BG*). The more the inducer excites and adapts a cone type along the x-axis in *panel a*, the less it responds to the constant background stimulus. In contrast, *panel d* shows that the adapted cone responds more along the y-axis the less inducers excite a single cone when moving from right to left along the x-axis. The second and third column represents inducers *(dashed lines)* and induced afterimages *(solid line)* in DKL- and CIELUV-space, respectively. Decreasing the intensity of a cone *(second row)* produces adaptation towards the directions opponent to the ones for increasing single cones *(first row)*. For comparison, the yellowish and light blue curves show the effects for inducer hues at 60 and 240 deg, which were the examples in *Figure 1* (main article) and *Figure S11* (models in DKL). When increasing CIELUV chroma from 0 to 100, the hue of the induced afterimage is bent towards the least adapted cone (cf. panel *e* or *f*). The *S*-cone is least adapted for the yellowish 60-deg inducer, which excited *L*- and *M*- cones; the *L*-cone is least adapted for the blue 240-deg inducer, which excites *S*- and *M*-cones. This is why the yellow curve is bent towards the *S*-cone direction, and the light blue curve towards the *L*-cone direction.

Code S1 | Cone Adaptation

(a) Matlab function *lms2cc* converting cone excitations (lms) to cone contrasts (cone_contrasts)

```
function [cone_contrasts, cone_increments] = lms2cc(lms, lms0)
```

```
% cone contrasts = [dL/L0, dM/M0, dS/S0]
% cone increments = [L-L0, M-M0, S-S0] (Kaiser & Boynton, 1996, p.564)
% INPUT:
% lms   = cone excitations of the stimuli (L, M, S).
% lms0  = cone excitations of the adapting background (L0, M0, S0).
% OUTPUT:
% cone_contrasts
% 2020.09.16 [cw]

cone_increments = bsxfun(@minus,lms,lms0); % [L-L0, M-M0, S-S0]
cone_contrasts  = bsxfun(@rdivide,cone_increments,lms0); % [dL/L0, dM/M0, dS/S0]
```

(b) Matlab function *cc2lms* converting cone contrasts (cc) to cone excitations (lms)

```
function [lms, cone_increments] = cc2lms(cc, lms0)
% cone contrasts (cc)       = [dL/L0, dM/M0, dS/S0]
% cone increments (dLMS)    = [ccL*L0, ccM*M0, ccS*S0]
% cone excitations (LMS)    = [dL+L0, dM+M0, dS+S0]
% (cf. Kaiser & Boynton, 1996, p.564)
% INPUT:
% cc    = cone contrasts [dL/L0, dM/M0, dS/S0]
% lms0  = cone excitations of adapting stimulus (e.g., background)
% OUTPUT:
% lms   = one excitations of non-adapted stimuli
% cone_increments = cone increments (dLMS)
% 2025.04.15 [cw]

cone_increments = bsxfun(@times,cc, lms0); % dLMS = [ccL*L0, ccM*M0, ccS*S0]
lms = bsxfun(@plus,cone_increments, lms0); % lms = [dL+L0, dM+M0, dS+S0]
```

(c) Matlab function *afterimage_simulator* combining the above functions (a+b) to calculate afterimage predictions

```
function [induced_lms, cone_increments, cone_contrasts] =
afterimage_simulator(inducer_lms, bg_lms, weights, adapt_strength, probe_lms)
% 2023.01.19 [cw]

if nargin < 5
    if nargin < 4
        adapt_strength = [];
        if nargin < 3
            weights = [1 1 1];
            if nargin < 2
                close all; clc;
                example;
                return;
            end
        end
    end
    probe_lms = bg_lms;
end

if isempty(weights)
    weights = [1 1 1];
end
if isempty(adapt_strength)
    adapt_strength = 1;
end

% WEIGHT CONES (optional) -------------------------------------------------
% depending on (1) completeness vs partial adaptation; (2) relative
% adaptation of cones compared one to another
inducer_lms2 = bsxfun(@times, inducer_lms, weights);

% CALCULATE COLOUR OF PROBE RELATIVE TO INDUCER ---------------------------
% i.e. taking INDUCER as the point of adaptation.
cone_increments = bsxfun(@minus,probe_lms,inducer_lms2); % cone increments
% = [L-L0, M-M0, S-S0] (Kaiser & Boynton, 1996, p.564)
cone_contrasts = bsxfun(@rdivide, cone_increments,inducer_lms2); % cone contrasts
= [dL/L0, dM/M0, dS/S0]

% INDUCTION: Calculate the induced colour relative to the background ------
% i.e. taking the BG as the point of adaptation.

% ARBITRARY SCALING (ignore, leave at 1)
adapted_cone_contrasts = cone_contrasts*adapt_strength;

% MAIN (induction):
cone_increments = bsxfun(@times, adapted_cone_contrasts,bg_lms);
induced_lms = bsxfun(@plus, cone_increments,bg_lms);
```

2. Cone-Opponent Model (DKL)

The below reproduces formula A.4.8 from *Brainard (1996)*. Note that the *L*+M term of the *S-(L+M)* mechanisms is the same as luminance *lum* (highlighted red):

$$\begin{matrix} lum/k_{lum} \\ (L-M)/k_{L-M} \\ S-(L+M)/k_S \end{matrix} = \begin{bmatrix} 1 & 1 & 0 \\ 1 & -1*L0/M0 & 0 \\ -1 & -1 & (L0+M0)/S0 \end{bmatrix} * (\Delta L \quad \Delta M \quad \Delta S)$$

ΔL, ΔM, ΔS are cone increments, which are the differences between *LMS* and the adapting background *LMS0*. The factors *k* are scaling factors. They have been chosen so that all terms are relative to the adapting background *LMS0*. To link that formula to the one in the main article, the luminance *lum* and S−(L+M) is first divided by the adapting luminance k_{lum}= k_s = L0+M0, and L−M by $k_{L\text{-}M}$=L0. This results in:

$$\begin{matrix} lum \\ L-M \\ S-(L+M) \end{matrix} = \begin{bmatrix} 1/(L0+M0) & 1/(L0+M0) & 0 \\ 1/L0 & -1/M0 & 0 \\ -1/(L0+M0) & -1/(L0+M0) & 1/S0 \end{bmatrix} * (L-L0 \quad M-M0 \quad S-S0)$$

At isoluminance, the matrix can be simplified because *lum* is zero, as shown by computing the first row:

$$\frac{L-L0}{L0+M0} + \frac{M-M0}{L0+M0} + 0$$

$$= \frac{L+M-L0-M0}{L0+M0} = \frac{L+M}{L0+M0} - 1$$

Since *L*+M = *L*0+M0 when stimuli are isoluminant with the background:

$$\frac{L0+M0}{L0+M0} - 1 = 1 - 1 = 0$$

So, at isoluminance **zero** can be plugged in for lum and for the lum term of S–(L+M):

$$\begin{matrix} lum \\ L-M \\ S-(L+M) \end{matrix} = \begin{bmatrix} \mathbf{0} & \mathbf{0} & 0 \\ 1/L0 & -1/M0 & 0 \\ \mathbf{0} & \mathbf{0} & 1/S0 \end{bmatrix} * (L-L0 \quad M-M0 \quad S-S0)$$

If the denominators of the conversion matrix are now integrated with the cone increments, cone contrasts CC_L = (L–L0)/L0, CC_M = (M–M0)/M0, *and* CC_S = (S–S0)/S0 are produced on the right (in red):

$$\begin{matrix} lum \\ L-M \\ S-(L+M) \end{matrix} = \begin{bmatrix} 0 & 0 & 0 \\ 1 & -1 & 0 \\ 0 & 0 & 1 \end{bmatrix} * (CC_L \; CC_M \; CC_S)$$

In the main article, *L*−M and S−(L+M) are called DKL_{L-M} and DKL_S, and *lum* is not mentioned because it is constant at 0. The adaptation model uses this same formula to calculate adaptation along the chromatic axes as $iDKL_{L-M}$ and $iDKL_S$.

To create the stimuli of *Experiment 2b*, L–M and S axes have been set to be maximal within the monitor gamut using the following determination of scaling factors; a Matlab function implementing the scaling is provided as part of *Code 2* below. To do this, unit length of the original axes of DKL has been determined as vectors with values of one in each direction of the axes:

$$L-M = [0 \quad 1 \quad 0]$$

$$M-L = [0 \quad -1 \quad 0]$$

$$S-(L+M) = [0 \quad 0 \quad 1]$$

$$(L+M)-S = [0 \quad 0 \quad -1]$$

These were converted to *RGB* via *LMS* using the above conversion from LMS increments (differences from adapting background) to DKL. The two conversions from DKL to LMS increments, *dLMS*, and then to *RGB* could be done in one single step, using an integrated matrix, which might be mathematically more elegant. However, here it is done step by step for the sake of explaining the logical steps. So, here is the conversion of unit vectors from DKL back to LMS:

$$dLMS_{unit} = [\frac{DKL_{unit}}{lms2dkl^T}]$$

Where DKL_{unit} are the four vectors at unit length and *lms2dkl* is the conversion matrix developed above, with *lum*, L–M and S–(L+M) as rows and *L*, M, and S as columns. Superscript *T* indicates that matrix *lms2dkl* is transposed. We divide through *lms2dkl* as this is a

back-transformation. From there LMS increments are converted to RGB increments using a matrix *rgb2lms* that lists the LMS signal of each monitor primary at maximum:

$$lms2rgb = \begin{bmatrix} L_R & M_R & S_R \\ L_G & M_G & S_G \\ L_B & M_B & S_B \end{bmatrix}$$

The conversion consists of dividing the LMS unit vectors, LMS_{unit} by the *lms2rgb* matrix:

$$dRGB_{unit} = [\frac{LMS_{unit}}{rgb2lms}]$$

Where $dRGB_{unit}$ contains the RGB unit increments for both directions of the *L*–M axis (*L*–M and M–*L*), and *S*–(*L*+M) axis (*S*–(*L*+M) and (*L*+M)–*S*). We then determine how often this RGB unit fits between the adapting background and the minimum and maximum RGB values. Minimum RGB values are 0, and maxima are 1 because the above conversions imply normalised and linear RGB values (not gamma distributed). So, the difference vectors between the adapting background, *RGB0*, and zero, and between adapting background and 1 correspond to the RGB gamut along the axes (in both directions). Dividing those gamuts by the RGB units provides the scaling factors for each primary separately. This is illustrated with the example of the L–M axis:

$$max_{L-M} = \frac{1 - RGB0}{L - M_{unit}}$$

And:

$$max_{M-L} = \frac{0 - RGB0}{M - L_{unit}}$$

The vectors max_{L-M} and max_{M-L} contain each a ratio for each primary *R*, G, and *B*, respectively. As the linear RGB values change linearly along the axis, the primary that is closest to the white-point/background in terms of the number unit vectors determines the maximum length of the axis. This is the primary with the lower absolute ratio.

$$maxLM = \min{(max_{L-M}, max_{M-L})}$$

The same formulas are applied to the *S*–(*L*+M) axis resulting in *maxS*. The respective axes in the transformation matrix *lms2dkl* are divided by those ratios, max_{LM} and max_S:

$$L - M = \frac{L - M}{max_{LM}}$$

And:

$$S - (L + M) = \frac{S - (L + M)}{max_S}$$

Where *L*–M and *S*–(*L*+M) correspond to the second and third row of matrix *lms2dkl*, respectively. Dividing the rows of the matrix by the scaling factor allows for direct conversion to a DKL space that is scaled according to monitor gamut.

Code S2 | Cone Opponency

(a) Matlab function *lms2dkl* converting cone excitations (lms) to cone-opponent DKL-space (dkl)

```
function [dkl, M] = lms2dkl(lms, lms0, monlms)
% Converts cone excitations (lms) to DKL coordinates (dkl) relative to the
% adapting white-point (lms0). Optionally, DKL space may be rescaled to fit
% into the monitor gamut (monlms).
% INPUT:
% lms    = cone excitations (in columns) of the stimuli (in rows)
% lms0   = cone excitations of the adapting stimulus (e.g., the background)
% monlms = L, M, S (in columns) for the monitor primaries R, G, B (in rows)
% OUTPUT:
% dkl   = DKL axis (in columns) for each lms stimulus (in rows).
% M     = transformation matrix that converts from lms to DKL.
% The transformation matrix M is equivalent to the one from Brainard
% (1996), except for the scaling. Input monlms rescales the axis so that
% the isoluminant hue circle fits into the monitor gamut; this is the same
% scaling as in DKLDemo of Psychtoolbox.
% 2025.04.11

% INCREMENTS:
dlms = lms-ones(size(lms,1),1)*lms0;

L0 = lms0(1);
M0 = lms0(2);
```

```
S0 = lms0(3);

% TRANSFORMATION MATRIX THAT SCALES RELATIVE TO WP (L0, M0, S0)
M = [...
    1/(L0+M0),  1/(L0+M0),      0;...
    1/L0,       -1/M0,      0;...
    -1/(L0+M0), -1/(L0+M0), 1/S0];

% RESCALING TO MONITOR GAMUT
if nargin > 2
    [maxL, maxS] = map2mongamut(M, lms0, monlms);
    M(2,:) = M(2,:)/maxL;
    M(3,:) = M(3,:)/maxS;
end

% APPLY TRANSFORMATION MATRIX
dkl = dlms * M';
```

(b) Matlab function *map2mongamut* rescaling DKL-axes to fit monitor gamut

```
function [maxLM, maxS] = map2mongamut(M, lms0, monlms)
% Produces scaling factors for the L-M and S-(L+M) axis. This function is
% based on David Brainard's function MaximizeGamutContrast in Psychtoolbox.
% INPUT:
% M = LMS to DKL conversion matrix, as implemented in function lms2dkl.
% lms0 = LMS signal (cone excitations) of the adapting white-point (i.e.,
% background).
% monlms = LMS of the monitor primaries (at maximum).
% OUTPUT:
% maxLM, maxS = Maximum within gamut expressed relative to the original
% dkl unit; dividing unit length by these maxima makes units the length of
% those maxima.
% 2025.07.11 based on PTB MaximizeGamutContrast. [cw]

% DEFINE THE POLES IN DKL AT UNIT LENGTH ----------------------------------
pols = [...
    0  1  0;... L-M pole
    0 -1  0;... M-L pole
    0  0  1;... S-(L+M) pole
    0  0 -1;... (L+M)-S pole
    ];

% CONVERT DKL TO CONE INCREMENTS (dlms) -----------------------------------
% Cone increments = lms signal - lms0;
pole_dlms = pols / M';

% CONVERT LMS TO RGB -------------------------------------------------------
% This can be done directly with the cone-increments:
rgb0 = lms0/(monlms(1:3,1:3));
pole_drgb = pole_dlms/(monlms(1:3,1:3));

% CALCULATE THE RATIO BETWEEN DKL UNIT LENGTH AND MAX RGB -----------------
% RGB gamut is 0 (minimum) and 1 (maximum); subtracting the rgb0 (adapting
% wp) allows determing the length away from rgb0. The result of this
% calculation indicates for each primary how many time the original DKL
% unit fits within the gamut of that primary.

% L-M Mechanism:
maxL = (1-rgb0)./(pole_drgb(1,:)); % L-M direction
maxM = (0-rgb0)./(pole_drgb(2,:)); % M-L direction

% S-(L+M) Mechanism:
maxS1 = (1-rgb0)./(pole_drgb(3,:)); % +S direction
maxS2 = (0-rgb0)./(pole_drgb(4,:)); % -S direction

% IDENTIFY SCALING FACTORS ------------------------------------------------
% The primary that defines the limit in a given direction is the smallest
% ratio because that primary would be the first to fall out of gamut when
% increasing DKL saturation. As both direction of the axes are scaled by
% the same factor, the scaling factor is the minimum length (absolute min)
% of both directions together.

maxLM = min(abs([maxL,maxM]));
maxS = min(abs([maxS1,maxS2]));
```

3. CIELUV

For high levels of lightness ($Y > 0.0089 * Y0$), the CIELUV formula for lightness L^* is:

$$L^* = 116 * (Y/Y0)^{1/3} - 16$$

where Y is the luminance of the stimulus and $Y0$ is the luminance of the adapting white-point. As $Y0$ and Y are constant in the present experiments (isoluminance), L^* is also constant. Chromatic coordinates u^* (green-red) and v^* (blue-yellow) are calculated as:

$$u^* = 13 * L^* * (u' - u0')$$

$$v^* = 13 * L^* * (v' - v0')$$

As $13 * L^*$ is constant, they only scale u^* and v^*, and the transformation depends only on ($u' - u0'$) and ($v' - v0'$). The coordinates u' and v' are the uniform chromaticity coordinates of the colour stimulus, and $u0'$ and $v0'$ are those for the adapting white-point. Here, adaptation is modelled by the subtraction ("Judd transform") of the chromaticity coordinates $u0'$ and $v0'$ (highlighted red). Unlike divisive von Kries adaptation, this is subtractive adaptation. The only terms that change for isoluminant stimuli are the chromaticity coordinates ($u' - u0'$) and ($v' - v0'$), which are calculated as:

$$u' = 4 * X/(X + 15 * Y + 3 * Z)$$

$$v' = 9 * Y/(X + 15 * Y + 3 * Z)$$

Importantly, the denominator (in red) is the same for u' and v'. This means that u' and v' are *projective transformations*, or *homographic projections*, of the Tristimulus values, X, Y, and Z. The projection can be conceived by expressing the transformation in *homogenous coordinates* (u' v' 1):

$$\begin{pmatrix} u' \\ v' \\ 1 \end{pmatrix} = \begin{pmatrix} 4 & 0 & 0 \\ 0 & 0 & 9 * Y0 \\ 1 & 3 & 15 * Y0 \end{pmatrix} * \begin{pmatrix} X \\ Z \\ 1 \end{pmatrix}$$

Dividing by $X{+}3Z{+}15Y0$ will restore the Euclidean plane. So, lines that are straight in Tristimulus space, remain straight in the uniform chromaticity diagram. As Y0 (luminance) is constant at isoluminace, this projection happens in the (X, Z) plane. The (X, Z) plane is a linear transformation of DKL-space. For this reason, lines that are straight in DKL-space remain straight in CIELUV space, and opponent hues (180° rotated) are the same in DKL- and CIELUV-space (cf. *Figure 1.c*). This, the conservation of straight lines, is also true for any linear transformation of XYZ, including linear (gamma-corrected) HSV- and RGB-space, and for chromaticity coordinates (x, y), which are also projective transformations of XYZ *(chapter 3 in Fairchild, 2013)*.

4. CIELAB

Lightness L^* in CIELAB is the same as in CIELUV. In CIELAB, chromatic coordinates depend on the same luminance condition *(Y/Y0)* as lightness L^*: $Y > 0.0089 * Y0$. For this luminance ratio, chromatic coordinates, a^* (green-red) and b^* (blue-yellow), are calculated as:

$$a^* = 500 * \left[(X/X0)^{1/3} - (Y/Y0)^{1/3}\right]$$

$$b^* = 200 * \left[(Y/Y0)^{1/3} - (Z/Z0)^{1/3}\right]$$

A divisive von-Kries-like adaptation transform is part of this formula (highlighted red). However, this transformation is calculated with the Tristimulus values *(X, Y, Z)* rather than the cone excitations *(L, M, S)*. This is why we may call it a "pseudo von-Kries transform" *(Fairchild, 2013, section 10.5)*. After calculating the adaptation ("post-adaptation"), the third root is taken of the adapted Tristimulus values. This post-adaptation transform only matters for representation in CIELAB space. As the same CIELAB values are converted back to X, Y, Z, the nonlinear post-adaptation transform is reverted. For this reason, post-adaptation transform is irrelevant for the modelling in this study. The reason for the difference between the predictions of the cone-adaptation model in *Figure 1.b* and those of the CIELAB model in *Figure 1.d* is only the pseudo von-Kries transform.

5. CIECAM02

CIECAM02 involves a complicated transformation that takes into account not only the immediate background of the stimulus that defines the adapting white-point (as in CIELUV and CIELAB), but also the intensity of the global surround. The surround intensity has an impact on the strength of adaptation. In this study, the surround is the dark experimental room. A dark surround would imply weak adaptation. However, adaptation was matched to produce induction effects of the same intensity as those in the other models. Assuming other surrounds does not affect the results, apart from the strength of induction, which is compensated for by using *z*-scores when comparing chroma across models (*Figure 2.j*). The first step of the transformation from Tristimulus values (X, Y, Z) to CIECAM02 involves a conversion of Tristimulus values to standardized cone-like primaries (*R*, *G*, *B*) based on Judd's linear transformation matrix. Although these primaries are more similar to cone excitations than Tristimulus values (as in CIELAB), they are still different from

cone excitations, especially R and G (*see Figure 16.9 in Fairchild, 2013*). That is why it makes sense to call them RGB rather than LMS. Then, a von-Kries-like adaptation transform is applied to those RGB primaries as follows:

$$rR = \left[\left(Y0 * \frac{D}{R0}\right) + (1 - D)\right] * R$$

$$rG = \left[\left(Y0 * \frac{D}{G0}\right) + (1 - D)\right] * G$$

$$rB = \left[\left(Y0 * \frac{D}{B0}\right) + (1 - D)\right] * B$$

Where R, G, and B are the original primaries, and the resulting rR, rG, and rB are the adapted ("relative") primaries. $Y0$ is, as in previous sections, the luminance of the white-point. D is a constant that indicates the degree of adaptation (1 = full adaptation, 0 = no adaptation) and depends on the surround intensity (see above). As mentioned above (see surround), using different values for D did not change the results of this study. So, D was set to 1 for full adaptation. Instead, the strength of afterimage predictions was controlled by downscaling the inducer chroma (to comparison colours in *Exp 1* or grand average in *Exp 2*) like with the other models. By doing this, the adaptation transform simplifies to a von-Kries adaptation of the *RGB* primaries:

$$rL = Y0 * \frac{R}{R0}$$

$$rM = Y0 * \frac{G}{G0}$$

$$rS = Y0 * \frac{B}{B0}$$

Multiplication by white-point luminance, $Y0$, is necessary because the XYZ and RGB are normalised. The von-Kries-like adaptation explains why the CIECAM02 model in *Figure 1.d* resembles the cone-adaptation model in *Figure 1.b*. After the adaptation transform, CIECAM02 applies post-adaptation nonlinear compressions to the adapted rR, rG, and rB. These are very complex but have roughly similar effects as square root functions (*cf. section 16.3 in Fairchild, 2013*). As for CIELAB, these transformations are irrelevant to the modelling in this study as they do not involve an effect of the adaptation colour, i.e., the inducer. Therefore, the differences between the CIECAM02 model (*Figure 1.d*) and the cone adaptation model (*Figure 1.b*) result from the difference between the *RGB* primaries used for CIECAM02 and proper cone excitations (*Figure 16.9 in Fairchild, 2013*).

6. Munsell-Opponent Model

The *Munsell system* consists of discrete surface colours, the *Munsell chips*, that had been arranged along 3 dimensions (*value*, hue, and chroma, where *value* is lightness) based on colour appearance judgements (*chapter 5 in Fairchild, 2013*). The discreteness and the illuminant-independent specification posed a challenge for identifying exact opponent colours of the inducers in the Munsell system. For precise predictions that can be compared with other models, continuous Munsell coordinates were needed. As continuous coordinates do not exist as real Munsell chips, the computed continuous Munsell coordinates are called "virtual" in the present context. The continuous coordinates had to be interpolated by the following approach:

(1) To determine Munsell coordinates for the inducers used in this study, CIELUV and DKL inducers have first been converted to CIELAB using the white-point of this study (monitor white).

(2) Munsell chips closest to inducers were identified in CIELAB. First, the two nearest lightness levels (L*) corresponding to Munsell chips were identified, then the two adjacent hue and chroma of Munsell chips at those lightness levels. This resulted in 8 points in CIELAB: 2 lightness levels (L*) x 2 chroma levels x 2 hues that corresponded with discrete Munsell coordinates of Munsell chips.

(3) Virtual continuous Munsell coordinates for an exact match were interpolated between those 8 closest Munsell chips. For this, the Munsell value, chroma, and hue of the inducer were linearly interpolated between those 8 Munsell coordinates.

(4) The virtual Munsell coordinates in the opposite hue direction were computed by shifting Munsell hue to the direction opposite to the inducer hue. As Munsell hues are defined by circular 40 hue steps, the opponent hue in Munsell space was determined as 20 steps away from the inducer Munsell hue. Munsell value and chroma of the opponent colour were kept the same as for the inducer.

The resulting opponent Munsell coordinates were then represented in CIELAB and from there transformed to the colour spaces used in the study (CIELUV, DKL). To represent the opponent Munsell coordinates in CIELAB, the above interpolation procedure was reversed:

(5) CIELAB coordinates of eight adjacent Munsell chips were identified for the opponent continuous Munsell coordinates.

(6) The exact CIELAB coordinates were linearly interpolated in CIELAB based on those adjacent chips.

(7) Those Munsell-opponent CIELAB values were then converted to CIELUV using the white-point of this study for comparison with other results.

This approach produces the red curve in *Figure 1.e*. In this approach, the CIELAB metric is only necessary and used for the linear interpolation based on adjacent chips, which concerns only very small colour differences. To obtain CIELAB values, the Munsell renotation table from *Newhall et al. (1943)* has been used. The CIELAB coordinates from the *Munsell Lab* of the *Rochester Institute of Technology* resulted from converting the original *xyY* of the renotation table to CIELAB space using *Illuminant* C to account for the colours being surfaces (*Munsell chips*). In general, CIELAB is used rather than CIELUV, because the divisive CIELAB adaptation transform is superior to the subtractive CIELUV transform in predicting colours across illuminant changes. As also evident in this study, the pseudo-von-Kries transform in CIELAB is not perfect in predicting adaptation across white-points. However, the white-points in this study had similar chromaticities (*Experiment 1*: x = 0.3304, y = 0.3526; see *Table S4* for *Experiment 2*) as *illuminant* C (x = 0.3101, y = 0.3162), implying that that any insufficiencies of the CIELAB transform are negligibly small in this context.

7. Hering-Opponent Model

The prototype adjustment task *(see section A.4)* yielded average typical hues in CIELUV at 10.6 deg for *red*, 72.2 deg for *yellow*, 125.1 deg for *green* and at 227.9 deg for *blue (cf. Table S3)*. These hue directions are similar to the unique hues measured by *Witzel & Gegenfurtner (2018, Figure 1)* and *Witzel et al. (2019, Table S2)*, as discussed above *(section A.4)*. They were thus taken as estimates of unique red, yellow, green, and blue and as the directions for the green-vs-red (10.6 vs 125.1 deg) and blue-vs-yellow (227.9 vs 72.2 deg) axes of the Hering-opponent model. CIELUV azimuths in between those Hering-axes were linearly interpolated to produce a proportional distance between the adjacent axes and carve out four new quadrants lying in between the red-yellow, yellow-green, green-blue, and blue-yellow directions. As a result, hues in CIELUV are compressed when Hering axes were closer than 90 degrees in CIELUV. This was the case between red and yellow, which differ by 61.6 deg, and between yellow and green, which differ by only 52.9 deg in CIELUV. In contrast, hues were stretched out between blue and red, which differed by 142.7 deg, and between green and blue, which differed by 102.8 deg. These compressions and stretches can be seen from the density of the black dots in *Figure 1.e* in the main article. Those black dots represent colours opponent in Hering coordinates for inducer hues that were equally distant in CIELUV.

To calculate opponent colours in Hering coordinates, the inducer was first expressed as a proportion of the difference between two adjacent unique hues. For examples, an inducer of 60 deg in CIELUV lies in the red-yellow quadrant. It therefore corresponds to:

(1) (60 deg – *red*) / (*yellow* – *red*) = 60deg – 10.6 deg / 61.6 deg = 80.2%

To obtain the opponent hue, this proportion of the inducer hue would be applied to the quadrant opposite to the inducer quadrant. In the example, that would be 80.2% away from green in the green-blue quadrant. This corresponds to:

(2) *green* + 80.2% * (*blue* – *green*) = 125.1 deg + 0.802 *102.8deg = 207.5 deg

Pulling these 2 steps together in one equation that converts inducers into proportional coordinates *RYGB* for Hering-opponent colours:

$$RYGB = UH3 + \frac{inducer - UH1}{UH2 - UH1} * (UH4 - UH3)$$

where all variables are in degree azimuth in CIELUV; *UH1* and *UH2* are the unique hues with an azimuth below (clockwise) and above (counterclockwise) the inducer hue; *UH3* and *UH4* are the lower and upper unique hues of the opposite quadrant. Differences are circular differences. This approach yielded the hues represented by black dots in *Figure 1.e*. These dots form a circle because Hering opponency does not make a prediction for chroma. To illustrate the hue distribution in *Figure 1.e*, the radius has been set to the same constant radius as the CIELUV model in Figure 1.c (black circle) but was excluded for the analyses of chroma visualised in *Figure 2.j*.

<end>
