# Supplementary material for: The non-opponent nature of colour afterimages
Source: Commun Psychol. 2025 Nov 1;3:154. doi: 10.1038/s44271-025-00331-5 (PMC12579601; doi:10.1038/s44271-025-00331-5)
Supplement: Supplementary file 3 — Description of Additional Supplementary Files [file 44271_2025_331_MOESM3_ESM.pdf]

# Description of Additional Supplementary Files

File name- Supplementary Movie 1

File description – Supplementary Movie 1 illustrates the afterimage colour for a yellowish inducer with a CIELUV hue of 60 degrees; the afterimage is expected to appear in the purplish hue at 256 degrees predicted by cone-adaptation (upper half-disk) rather than the bluish hue at 240 degrees predicted by cone-opponency (lower half-disk).

File name- Supplementary Movie 2

File description – Supplementary Movie 2 illustrates the afterimage colour for a blue inducer with a CIELUV hue of 240 degrees; the afterimage is expected to appear in the reddish brown at 46 degrees predicted by cone-adaptation (upper half-disk) rather than the yellowish brown at 60 degrees predicted by cone-opponency (lower half-disk).

File name- Supplementary Movie 3

File description – Supplementary Movie 3 illustrates the afterimage colour for a pink inducer with a CIELUV hue of 340 degrees; the afterimage is expected to appear in the green at 160 degrees predicted by both cone-adaptation (upper half-disk) and cone-opponency (lower half-disk).

File name- Supplementary Movie 4

File description – Supplementary Movie 4 illustrates the afterimage colour for a green inducer with a CIELUV hue of 100 degrees; the afterimage is expected to appear in the purple at 280 degrees predicted by both cone-adaptation (upper half-disk) and cone-opponency (lower half-disk).
