# Supplementary figures and images for: The non-opponent nature of colour afterimages
Source: Commun Psychol. 2025 Nov 1;3:154. doi: 10.1038/s44271-025-00331-5 (PMC12579601; doi:10.1038/s44271-025-00331-5)

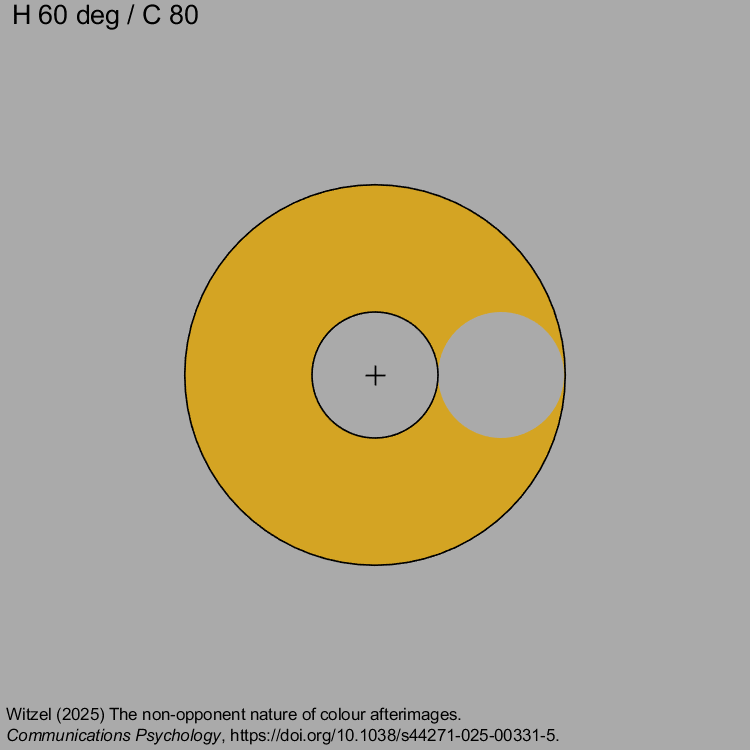

Supplement: Supplementary file 4 — Supplementary Movie 1 [file 44271_2025_331_MOESM4_ESM.gif]

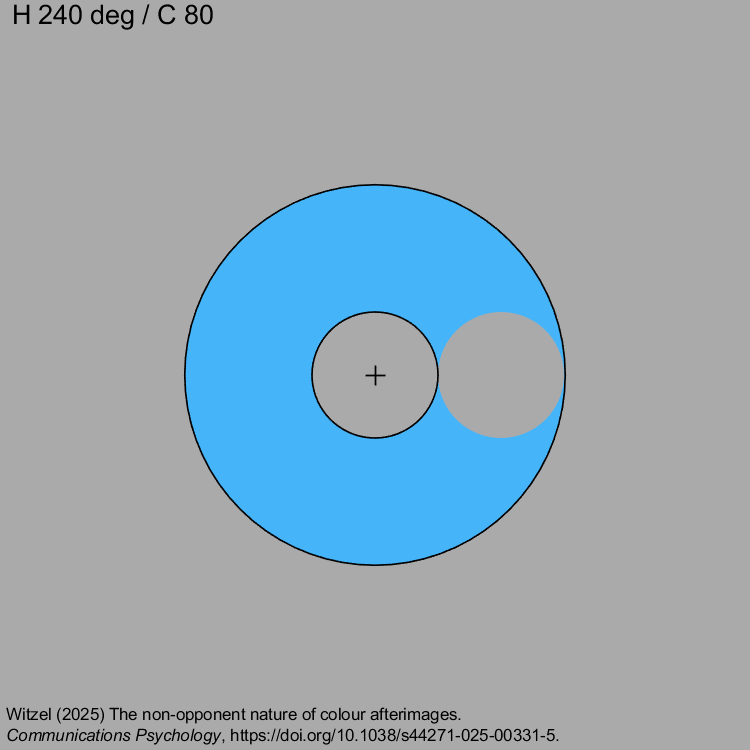

Supplement: Supplementary file 5 — Supplementary Movie 2 [file 44271_2025_331_MOESM5_ESM.gif]

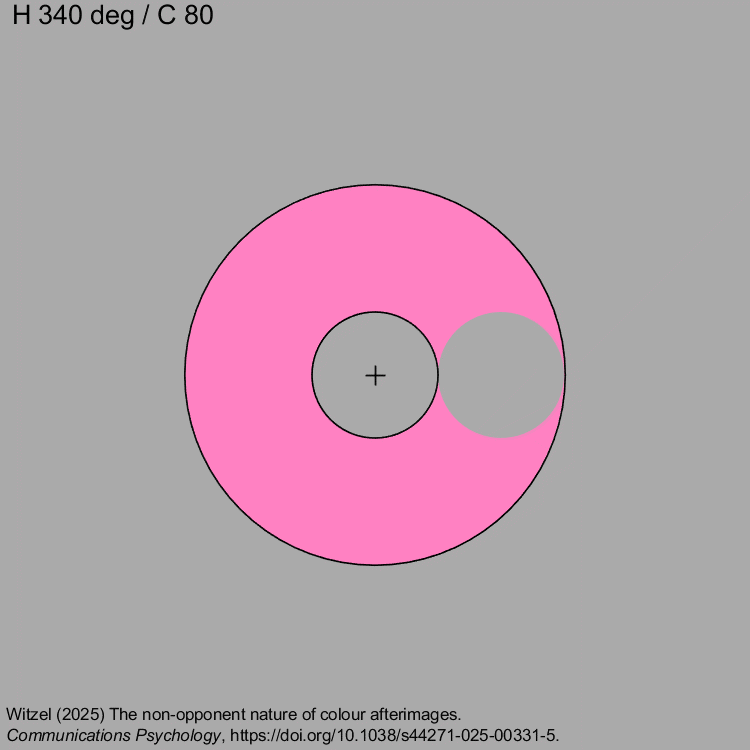

Supplement: Supplementary file 6 — Supplementary Movie 3 [file 44271_2025_331_MOESM6_ESM.gif]

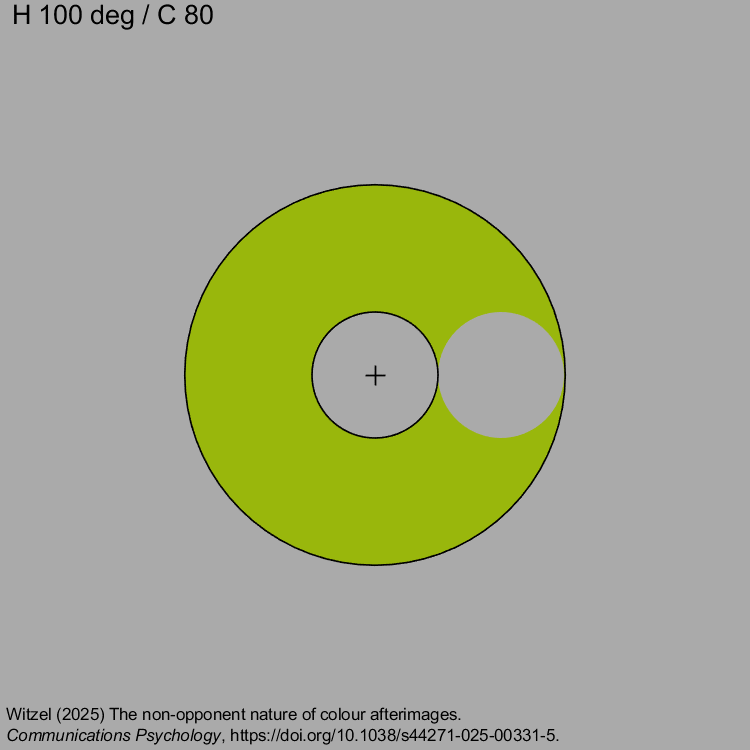

Supplement: Supplementary file 7 — Supplementary Movie 4 [file 44271_2025_331_MOESM7_ESM.gif]
