# Supplementary material for: The non-opponent nature of colour afterimages
Source: Commun Psychol. 2025 Nov 1;3:154. doi: 10.1038/s44271-025-00331-5 (PMC12579601; doi:10.1038/s44271-025-00331-5)
Supplement: Supplementary file 8 — Reporting summary [file 44271_2025_331_MOESM8_ESM.pdf]

nature portfolio

Corresponding author(s): Christoph Witzel

Last updated by author(s): Sep 8, 2025

Reporting Summary

Nature Portfolio wishes to improve the reproducibility of the work that we publish. This form provides structure for consistency and transparency in reporting. For further information on Nature Portfolio policies, see our Editorial Policies and the Editorial Policy Checklist.

Statistics

For all statistical analyses, confirm that the following items are present in the figure legend, table legend, main text, or Methods section.

n/a	Confirmed	
☐	☒	The exact sample size (*n*) for each experimental group/condition, given as a discrete number and unit of measurement
☐	☒	A statement on whether measurements were taken from distinct samples or whether the same sample was measured repeatedly
☐	☒	The statistical test(s) used AND whether they are one- or two-sided<br>*Only common tests should be described solely by name; describe more complex techniques in the Methods section.*
☐	☒	A description of all covariates tested
☐	☒	A description of any assumptions or corrections, such as tests of normality and adjustment for multiple comparisons
☐	☒	A full description of the statistical parameters including central tendency (e.g. means) or other basic estimates (e.g. regression coefficient) AND variation (e.g. standard deviation) or associated estimates of uncertainty (e.g. confidence intervals)
☐	☒	For null hypothesis testing, the test statistic (e.g. F, t, r) with confidence intervals, effect sizes, degrees of freedom and P value noted<br>*Give P values as exact values whenever suitable.*
☒	☐	For Bayesian analysis, information on the choice of priors and Markov chain Monte Carlo settings
☒	☐	For hierarchical and complex designs, identification of the appropriate level for tests and full reporting of outcomes
☐	☒	Estimates of effect sizes (e.g. Cohen's *d*, Pearson's *r*), indicating how they were calculated

Our web collection on statistics for biologists contains articles on many of the points above.

Software and code

Policy information about availability of computer code

Data collection	Experiments were programmed with the Psychtoolbox for Matlab.
Data analysis	Data analyses were done with Matlab.

For manuscripts utilizing custom algorithms or software that are central to the research but not yet described in published literature, software must be made available to editors and reviewers. We strongly encourage code deposition in a community repository (e.g. GitHub). See the Nature Portfolio guidelines for submitting code & software for further information.

Data

Policy information about availability of data

All manuscripts must include a data availability statement. This statement should provide the following information, where applicable:

- Accession codes, unique identifiers, or web links for publicly available datasets
- A description of any restrictions on data availability
- For clinical datasets or third party data, please ensure that the statement adheres to our policy

The data used for the analyses reported in this paper are available in a Zenodo repository at https://doi.org/10.5281/zenodo.13328099.

Human research participants

Policy information about studies involving human research participants and Sex and Gender in Research.

Reporting on sex and gender	Sex is reported, but not part of the study design.
Population characteristics	Averages and standard deviations of age are reported, but not part of the study design. Colour vision deficiencies were an exclusion criterion. This is all reported in the manuscript.
Recruitment	Participants were recruited through a covenience sample. Except for the author (identified as cw), participants were all naive about the models tested and the implications of the models for colour appearance. Even for the author, it was not possible to guess stimulus and response parameters from the colour adjustment. Thus, responses could not be affected by demand characteristics. The main challenge was to ascertain that naive participants followed the instructions of fixating the centre, as eye movements undermined adaptation and thus afterimages. As reported in the manuscript, some participants (cf. Figure S2, e.g., f2, f4, f5, f6) had thus weaker afterimages than others (cf. Figure 2, e.g. f1, f3, m3, f7). Although weaker afterimages involved more measurement noise, they still confirmed the main results as all individual data was in line with the aggregated results (cf. Figures S2-4).
Ethics oversight	Experiment 1: Ethics committee at the University of Gießen (LEK 2017-0030); Experiments 2-3: Faculty Ethics Committee at the University of Southampton (ERGO 65442).

Note that full information on the approval of the study protocol must also be provided in the manuscript.

Field-specific reporting

Please select the one below that is the best fit for your research. If you are not sure, read the appropriate sections before making your selection.

☒ Life sciences ☐ Behavioural & social sciences ☐ Ecological, evolutionary & environmental sciences

For a reference copy of the document with all sections, see nature.com/documents/nr-reporting-summary-flat.pdf

Life sciences study design

All studies must disclose on these points even when the disclosure is negative.

Sample size	Main analyses were conducted across stimulus samples. Experiment 1.b involved 24 stimuli. Experiment 2 double-checked results with a larger sample of 72 stimuli. Different strategies were used to obtain reliable data per stimulus and test different predictions derived from the same hypothesised models. The very first measurement (Experiment 1.a) involved 32 participants with 3 repeated measurements per stimulus; Experiment 1.b involved only 1 repetition per stimulus, but more participants (N = 52). Experiment 2 took a completely different approach, focusing on individual data, thus collecting 5 repeated measurements for each of 72 stimuli across 45 blocks. Results were replicated across 10 participants. Experiment 3 still increased the number of stimuli measured, as different levels of saturation (CIELUV chroma) were added. Results were replicated across 5 participants.
Data exclusions	In Experiment 2, data has been excluded for 3 participants who only tried out the task with 1 or 2 blocks, but did not want or follow up to complete data collection (thus, there was no data for the full set of 72 stimuli). To avoid data exclusion, two participants (m2 and m3) who provided data for all stimuli (9 hue sets), but dropped out before completing all five repeated measurements in Experiment 2 have been included. The number of blocks is reported in Figures S8-S10 (k = 1 and k = 3, in contrast to k = 5 for all other participants).
Replication	In each Experiment, different types of predictions were tested derived from the same hypothesised models. Thus, the consistent results across all measures and all experiments provide replications at the conceptual level (i.e., test of hypothesis). Test of specific predictions have also been replicated: Experiment 2 replicated the results of Experiment 1 with a different sample of participants, different task, and a different sampling strategy (high number of repeated measurments, instead of high number of participants). Experiment 2 also replicated all results for aggregated data across each of the 10 individual observers. Experiment 2.b replicated the results from Experiment 2.a with a different stimulus sample. The extensive measurements for Experiment 3 were replicated across 5 participants.
Randomization	Stimuli were randomised within blocks. Block identification and any link to the responses was unintelligible to participants.
Blinding	There was no group allocation. As said above, stimulus conditions and the meaning of responses were unintelligible to participants, this includes the author participant (cw).

Reporting for specific materials, systems and methods

We require information from authors about some types of materials, experimental systems and methods used in many studies. Here, indicate whether each material, system or method listed is relevant to your study. If you are not sure if a list item applies to your research, read the appropriate section before selecting a response.

Materials & experimental systems

n/a	Involved in the study
☒	☐ Antibodies
☒	☐ Eukaryotic cell lines
☒	☐ Palaeontology and archaeology
☒	☐ Animals and other organisms
☒	☐ Clinical data
☒	☐ Dual use research of concern

Methods

n/a	Involved in the study
☒	☐ ChIP-seq
☒	☐ Flow cytometry
☒	☐ MRI-based neuroimaging
